# The dynamic transcriptional and translational landscape of the model antibiotic producer *Streptomyces coelicolor* A3(2)

**DOI:** 10.1038/ncomms11605

**Published:** 2016-06-02

**Authors:** Yujin Jeong, Ji-Nu Kim, Min Woo Kim, Giselda Bucca, Suhyung Cho, Yeo Joon Yoon, Byung-Gee Kim, Jung-Hye Roe, Sun Chang Kim, Colin P. Smith, Byung-Kwan Cho

**Affiliations:** 1 Department of Biological Sciences, Korea Advanced Institute of Science and Technology, Daejeon 305-701, Korea; 2 KAIST Institute for the BioCentury, Korea Advanced Institute of Science and Technology, Daejeon 305-701, Korea; 3 School of Chemical and Biological Engineering, Seoul National University, Seoul 151-742, Korea; 4 Department of Microbial Sciences, Faculty of Health and Medical Sciences, University of Surrey, Surrey GU2 7XH, UK; 5 Department of Chemistry and Nano Science, Ewha Womans University, Seoul 120-750, Korea; 6 School of Biological Sciences, Seoul National University, Seoul 151-742, Korea; 7 Intelligent Synthetic Biology Center, Daejeon 305-701, Korea

## Abstract

Individual *Streptomyces* species have the genetic potential to produce a diverse array of natural products of commercial, medical and veterinary interest. However, these products are often not detectable under laboratory culture conditions. To harness their full biosynthetic potential, it is important to develop a detailed understanding of the regulatory networks that orchestrate their metabolism. Here we integrate nucleotide resolution genome-scale measurements of the transcriptome and translatome of *Streptomyces coelicolor*, the model antibiotic-producing actinomycete. Our systematic study determines 3,570 transcription start sites and identifies 230 small RNAs and a considerable proportion (∼21%) of leaderless mRNAs; this enables deduction of genome-wide promoter architecture. Ribosome profiling reveals that the translation efficiency of secondary metabolic genes is negatively correlated with transcription and that several key antibiotic regulatory genes are translationally induced at transition growth phase. These findings might facilitate the design of new approaches to antibiotic discovery and development.

## Abstract (web summary)

Bacteria of the genus *Streptomyces* produce a great variety of natural products, the biosynthesis of which is subject to complex regulatory networks. Here the authors present a high-resolution, genome-wide analysis of the transcriptome and translatome of *Streptomyces coelicolor* under various growth conditions.

## Graphical abstract

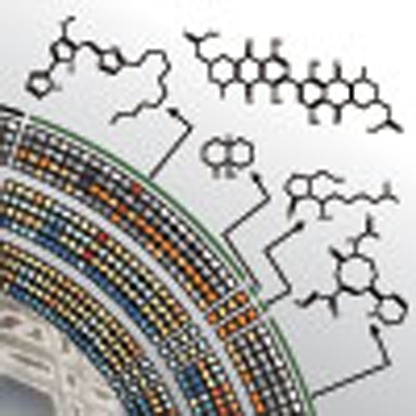

Recent advances in next-generation sequencing techniques have unveiled the previously under-estimated potential for secondary metabolite production in *Streptomyces* bacteria, known producers of more than 70% of antibiotics and other bioactive compounds used in medicine, agriculture and in the food industry^1^,^2^. As an example, the 8.7-Mb high G+C genome of the model *Streptomyces* species, *Streptomyces coelicolor*, encodes hundreds of secondary metabolic genes, which orchestrate the synthesis of various antibiotic compounds^3^,^4^. The biosynthesis of these metabolites often coincides with a physiological transition from primary to secondary metabolism and morphological differentiation. Both developmental switches are governed by highly interconnected regulatory mechanisms at transcriptional, translational and post-translational levels.

During the last two decades much progress has been made in the understanding of the molecular basis of antibiotic gene regulation, mainly focusing on the transcriptional changes in gene expression occurring at the physiological transition to antibiotic production. In recent years, a comprehensive array of genome-scale measurement techniques have been developed, mainly in bacterial systems to understand multi-layered and interconnected organizational components of bacterial genomes such as promoters, transcription start sites (TSSs) and regulatory small RNAs (sRNAs)^5^,^6^,^7^. Although expression at the level of transcription represents an important level of control, it is emerging that the extent of post-transcriptional regulation of gene expression in bacteria has been relatively underestimated. It is therefore important to determine which transcripts are being translated, through recently optimized techniques such as ‘ribosome profiling'; this method has been successfully used in prokaryotic and eukaryotic systems, where the quantification of such ribosome-protected fragments was shown to correlate well with cellular protein abundance^8^,^9^,^10^.

Systems-level analyses of unprecedented depth that integrate multiple genome-scale measurements have recently been reported for *Caulobacter crescentus*, and comprehensive transcriptional profiling of *Salmonella enterica* (including systematic TSS identification) has also been reported^10^,^11^. Here we describe the transcriptome and translatome in both primary and secondary metabolism of an antibiotic-producing bacterium, *S. coelicolor*, and we catalogue comprehensively the transcribed sequences and their associated TSSs within the genome.

## Results

### Landscape of primary transcriptome

**Figure 1:**
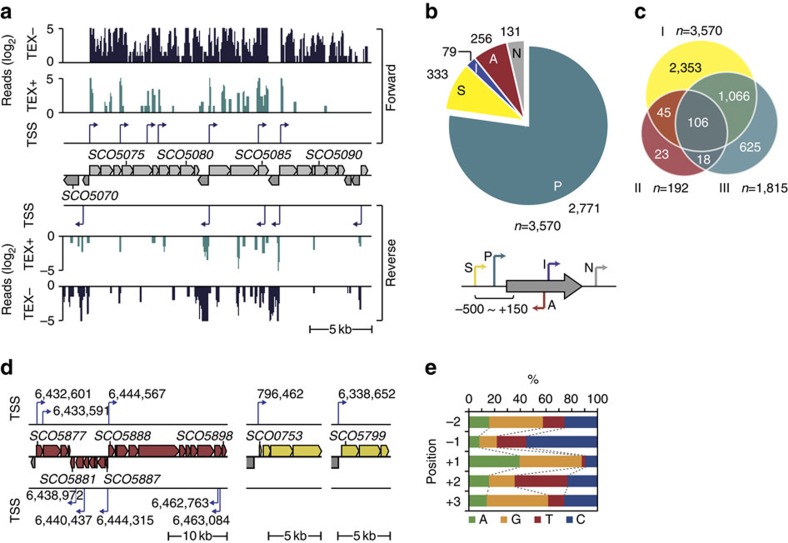
Determination of the transcriptional architecture of the *S. coelicolor* genome. (**a**) Example of a dRNA-seq profile mapped onto the *S. coelicolor* genome. For TSS determination, total RNA samples from 44 growth conditions were pooled and two sequencing libraries were constructed, one from TEX-treated (TEX+) and the other from untreated total RNA (TEX−); TEX, terminator-5′-phosphate-dependent exonuclease. The criteria for assigning TSS are detailed in the Methods. (**b**) A total of 3,570 TSSs were identified and classified according to their positions relative to adjacent open reading frames (ORFs). TSSs located from 500 bp upstream to 150 bp downstream of the respective annotated start codon of each ORF were classified as the primary (P) or secondary (S). TSSs located within an annotated ORF or on the opposite strand were classified as internal (I) or antisense (A), respectively. TSSs not included in any of these categories were classified as intergenic (N). (**c**) Mapped TSSs in relation to those reported from previous studies. I, this study; II, ref. 16.; III, ref. 18. (**d**) TSSs associated with secondary metabolic gene clusters; prodiginine (left), bacteriocin (middle) and siderophore (right). (**e**) Proportion of each nucleotide at TSS (+1) and 2 nt upstream and downstream of the TSS.

TSS maps of bacterial genomes allow the discovery of regulatory elements of gene expression, such as the regulatory sRNAs and 5′-untranslated regions (5′-UTRs) that regulate translational efficiency^12^,^13^,^14^,^15^. In order to catalogue these components, we mapped comprehensively the landscape of the whole set of newly synthesized RNAs (the primary transcriptome) of *S. coelicolor* A3(2) strain M145. 5′-Ends of primary transcripts were massively sequenced from mycelium that had been cultivated under a diverse range of growth conditions by using the differential RNA-sequencing (dRNA-seq) approach^7^,^16^. We designed a suite of 44 different growth conditions that reflect many of the environmental perturbations encountered by the bacterium, in order to maximize the chances of detection of the full repertoire of TSSs (Supplementary Table 1)^3^,^17^. As illustrated in Fig. 1a, the 5′-ends of primary transcripts were selectively determined (see the Methods for sequencing statistics and TSS mapping criteria), identifying a total of 3,570 TSSs (Supplementary Data 1).

These were further categorized by their positions relative to known coding sequences (Fig. 1b), giving 2,771 primary TSSs associated with currently annotated genes, which corresponds to 35.0% of the total genes in the *S. coelicolor* genome (note that the monocistronic and operonic structure have not been considered). 333 secondary TSSs were identified, which were detected in addition to the primary TSSs (see the Methods for detection criteria), revealing a total of 297 transcription units initiated by more than one TSS. A total of 256 TSSs mapped in the antisense strand of 241 genes, suggesting the presence of regulatory antisense sRNAs. A total of 79 internal TSSs were also detected, within 73 genes, and 131 TSSs mapped to intergenic regions with no previously associated genes. In total, 230 novel transcripts were predicted, 138 of which were represented as antisense transcripts and the others transcribed from intergenic regions. Of the 3,570 TSSs identified in the present study, 2,353 are reported here for the first time, whereas the other 1,217 of the TSSs were identified previously (Fig. 1c) (refs 16, 18); 666 TSSs reported in previous studies were not identified in this study and this discrepancy could be attributable to condition-specific expression from TSSs, because of the complex metabolism of the organism^6^,^17^.

Our cultivation conditions encompassed those appropriate to triggering secondary metabolism as 10 out of 11 secondary metabolic gene clusters that previously had identified TSSs were also identified in our study (Supplementary Table 2). In our study, a total of 68 TSSs were assigned to 18 of the 28 secondary metabolic gene clusters identified in the *S. coelicolor* genome (Supplementary Fig. 1) (ref. 1). For example, the biosynthesis of prodiginine is mediated via at least six TSSs in the upstream regions of SCO5877, SCO5878, SCO5881, SCO5882, SCO5887 and SCO5888 in the 30-kb biosynthetic gene cluster (Fig. 1d)^19^. Independent verification of the TSS mapping for the prodiginine cluster was obtained by 5′-rapid amplification of cDNA ends (Supplementary Fig. 2). Furthermore, we observed nine primary TSSs for putative secondary metabolic gene clusters, such as bacteriocin (genomic position: 796,462) and siderophore (genomic position: 6,338,652) (Fig. 1d and Supplementary Fig. 1). Although TSS mapping confirmed that *S. coelicolor* can use any nucleotide to initiate transcription, a purine is preferred in 87.9% of the cases (Fig. 1e). Interestingly, a pyrimidine is strongly preferred at the −1 (T, 22.7% and C, 55.5%) and +2 (T, 41.0% and C, 23.4%) positions, respectively. Based on the current *S. coelicolor* genome annotation, we have identified an average of 1 TSS for every 2.3 protein-coding genes, which approximates to more than one TSS per predicted transcription unit^20^. To evaluate reproducibility of TSS results, an independent dRNA-seq experiment was conducted with RNA from a single mid-exponential phase culture; the results demonstrated good concordance between a high proportion of the TSSs identified from this sample and the above analysis of the pooled RNA (Supplementary Fig. 3).

### Analysis of 5′ upstream sequences

**Figure 2:**
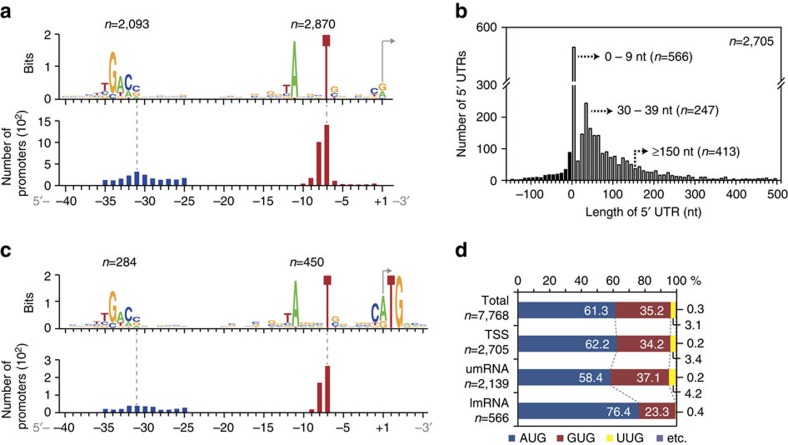
Genome-scale analysis of promoter sequences. (**a**) −10 motif (5′-TANNNT) and −35 motif (5′-NTGACC) were identified relative to TSS position (+1). The analysis showed three identical positions to the −10 motif of the *E. coli* promoter (that is, 5′-TATAAT) recognized by its housekeeping sigma factor (σ^70^). It has been suggested that the well-conserved TTG motif commonly found in the 5′ half of the *E. coli* −35 motif (that is, TTGACA) is located in the 5′ half of the *S. coelicolor* −35 motif^38^. Although *S. coelicolor* has a lower level of conservation of the TTG motif this analysis clearly identifies the motif at the same position. The bottom panel shows the position distribution of the −10 motif (red) and −35 motif (blue) relative to the TSS. (**b**) Distribution of 5′-UTR lengths reveals a dual peak distribution at 30–39 nt and 0–9 nt; the latter group are considered to produce leaderless mRNAs (lmRNAs). (**c**) The same −10 and −35 consensus sequences are observed upstream of lmRNAs. The motif around the TSS (+1) is also indicated. The third motif found at the +1 position clearly shows the translation initiation codon, indicating that lmRNAs are translated without 5′-UTR-mediated recognition. (**d**) Start codon usage of all open reading frames (ORFs; Total), primary TSS-identified ORFs (TSS), 5′-UTR-associated genes (umRNA) and leaderless genes (lmRNA).

The diverse sequences of *S. coelicolor* promoters must reflect, to some extent, the fact that its genome encodes >60 different sigma factors, contributing to its complex transcriptional patterns. To gain insight into the transcription efficiency of individual genes, it is important to identify the conserved promoter elements, such as the −10 and −35 sequences. The TSS positions enabled us to analyse the 5′-upstream sequence of each transcription unit. The conserved −10 motif (TANNNT) and less-conserved −35 motif (NTGACC) were identified in 80.4% (2,870 out of 3,570; *P*<0.05; MEME) and 58.6% (2,093 out of 3,570; *P*<0.05; MEME) of the identified TSSs, respectively (Fig. 2a and Supplementary Fig. 4). In addition to the variable internal NNN region of the −10 motif, which plays a critical role in promoter activity, the diversity in length of the spacer region probably reflects the sigma factor diversity, some of which are known to have different promoter spacing requirements (Supplementary Figs 5 and 6) (refs 21, 22).

The 5′-UTR of bacterial mRNAs typically contains a short motif called the Shine-Dalgarno sequence, which mediates ribosome binding and influences translational efficiency^23^. In addition to this, 5′-UTR sequences frequently contain additional regulatory sequences for post-transcriptional regulation^24^. From the primary TSSs of known coding sequences (2,705 in total), we calculated the distribution of 5′-UTR lengths; they have a median length of 44 nucleotides (nt) and the most frequent size range of 30–39 nt (Fig. 2b) (refs 7, 25, 26). Long leader sequences (length of 5′-UTR longer than 150 nt) were found in 413 transcripts (15.3%), suggesting the presence of potential regulatory RNA structures mediating post-transcriptional regulation^16^. Interestingly, 21.0% of primary TSSs produce leaderless mRNAs (lmRNAs) with the length of 5′-UTR shorter than 9 nt. The full list of lmRNAs is detailed in Supplementary Data 2 and the comparison with reported lmRNAs from other studies is summarized in Supplementary Fig. 7 (refs 16, 18, 25). The lmRNAs are transcribed from promoters that have similar consensus sequences to promoters for 5′-UTR-associated mRNA transcripts (umRNAs; Fig. 2c); the third motif found at the +1 position represents the translation initiation codon, suggesting the lmRNAs are translated without 5′-UTR-mediated recognition. AUG (58.4%) and GUG (37.1%) are almost exclusively used as the translation initiation codons for umRNAs, whereas AUG (76.4%) is highly preferred for lmRNAs (Fig. 2d). Functional categorization of genes encoded by lmRNAs (together with associated operonic genes, if the leaderless transcript is polycistronic) revealed that many are linked with the cell membrane and transcriptional regulation, particularly the TetR family regulators (Supplementary Data 2) (ref. 20). Five of the 49 primary TSSs assigned to the secondary metabolic gene clusters generate lmRNAs.

### Dynamic transcriptional landscape

Temporal alteration of cellular functions is achieved by the modulation of expression of a large array of genes. To elucidate the changes in expression levels of the individual genes, we used strand-specific RNA-seq (ssRNA-seq) that exploits the dUTP second-strand marking method (Supplementary Fig. 8a–c) (ref. 27). Four sampling times for *S. coelicolor* cultures grown in liquid R5− to mid-exponential, transition, late exponential and stationary phase were monitored. The onset of secondary metabolism was signalled by the appearance of the pigmented antibiotics produced by *S. coelicolor* at the transition growth phase (prodiginine) and during stationary phase (actinorhodin), respectively (Supplementary Fig. 9). As an example, ssRNA-seq of the prodiginine gene cluster shows a marked increase in expression between transition and late exponential growth phases (Supplementary Fig. 10) (ref. 28). The ssRNA-seq data were also used to determine the temporal modulation of expression of sRNAs identified from dRNA-seq (Supplementary Data 3). As an example, two sRNAs were observed from the antisense strand of SCO6762 (sRNA209) and the intergenic region between SCO3436 and SCO3437 (sRNA090), respectively (Supplementary Fig. 11).

**Figure 3:**
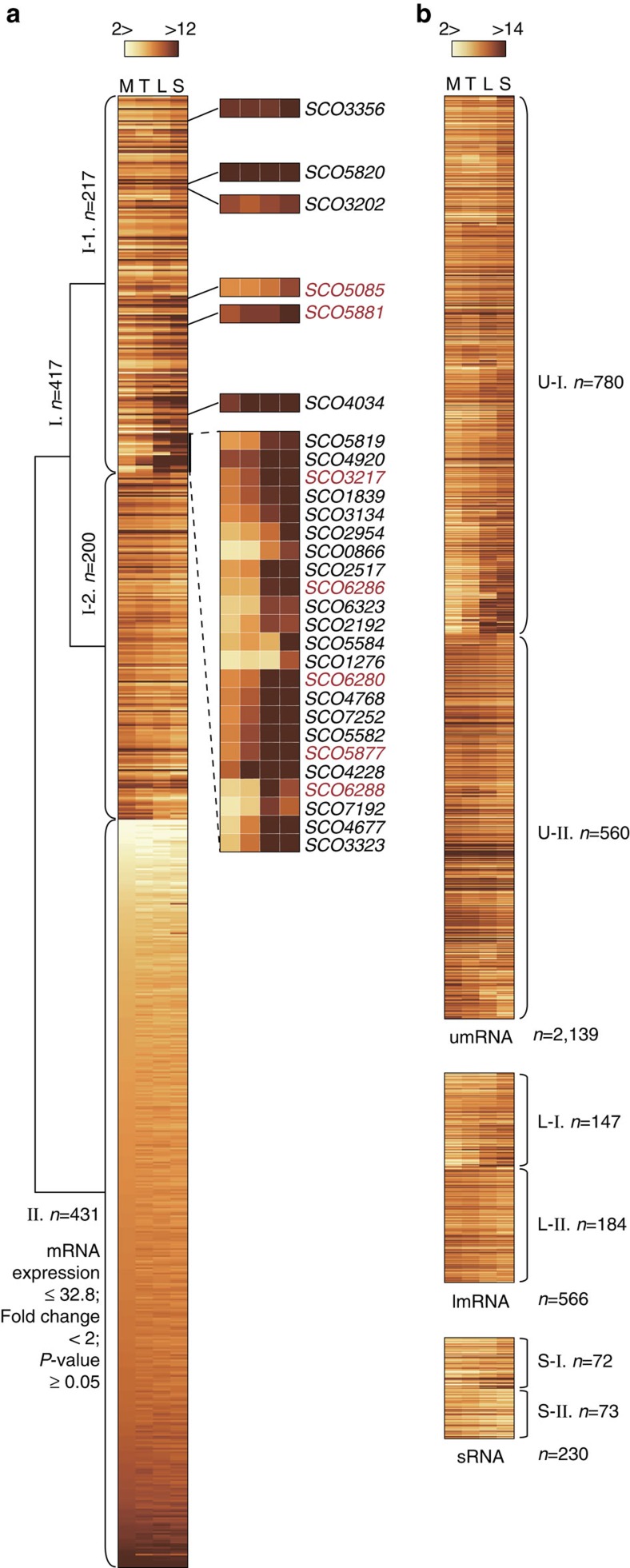
Transcriptome dynamics at different growth phases. (**a**) All transcriptional regulatory genes are clustered by their expression patterns where almost half of the regulatory genes are differentially expressed at different growth phases (Ι), whereas the other half showed no changes, or expression levels lower than the cutoff (ΙΙ). Bold black letters indicate sigma factors, and bold red letters indicate regulators of secondary metabolic gene clusters. (**b**) Differential expression of umRNAs, lmRNAs and sRNAs at different growth phases. Clusters are labelled U-I, U-II, L-I, L-II, S-I and S-II and the genes comprising each cluster are listed in Supplementary Data 6. M, mid-exponential phase; T, transition phase; L, late exponential phase; S, stationary phase.

We estimate that at least 6,000 genes are transcribed at the mid-exponential growth phase of *S. coelicolor* (Supplementary Data 4 and Supplementary Fig. 12). The most highly expressed genes under all four time points were SCO4655, SCO4729, SCO4762 and SCO4296, the first two encoding DNA-directed RNAP subunits and the second two encoding, respectively, the two chaperonin genes *groEL1* and *groEL2*. Among the highly expressed genes, a total of 4,558 showed twofold or greater changes in expression at two or more time points (DESeq *P*<0.05; Supplementary Data 5 and Supplementary Fig. 13a,b). We assessed growth phase-dependent changes in expression of all predicted regulatory genes, based upon the current genome annotation. Their expression patterns formed two large clusters in which almost equal numbers of regulatory genes were either differentially expressed (cluster Ι) or showed no or negligible changes in expression levels (cluster ΙΙ) relative to the growth phase (Fig. 3a and Supplementary Data 6). In cluster I, transcription of 417 regulatory genes showed growth phase-dependent induction or repression. This includes the antibiotic cluster-specific regulators SCO5085 (*actΙΙ-ORF4*), SCO5881 (*redZ*), SCO5877 (*redD*), SCO3217 (*cdaR*), SCO6280 (*kasO*) together with SCO6286, and SCO6288. The gene encoding the housekeeping σ-factor *hrdB* showed strong expression under all growth phases, whereas the expression levels of genes encoding alternative σ-factors such as *sigE*, *sigN* and *bldN* were significantly altered at different growth phases (Supplementary Data 6).

The dynamics of expression of the different transcript classes were compared, revealing higher median transcript levels for umRNAs at all growth conditions relative to those for lmRNAs (Wilcoxon rank-sum test *P*<2.6 × 10^−7^; Supplementary Fig. 14). The majority of lmRNAs and sRNAs (58.8% and 63.0%, respectively) displayed twofold or greater changes in their transcript level at two or more time points (*P*<0.05; Fig. 3b and Supplementary Data 6); similarly, 63.2% umRNAs were differentially expressed. Taken together, our integration of dRNA-seq and ssRNA-seq data measured from diverse sets of culture conditions provides a comprehensive and quantitative picture of the transcriptional landscape of *S. coelicolor*. Furthermore, independent quantitative PCR analysis was conducted on RNA from independent cultures at different growth phases using a selection of genes that represent a broad range of expression levels; the comparison of the ssRNA-seq data and quantitative PCR results showed a *R*^2^ value of 0.92 demonstrating the validity and reproducibility of the ssRNA-seq-based quantification (Supplementary Fig. 15).

### Dynamic translational landscape

**Figure 4:**
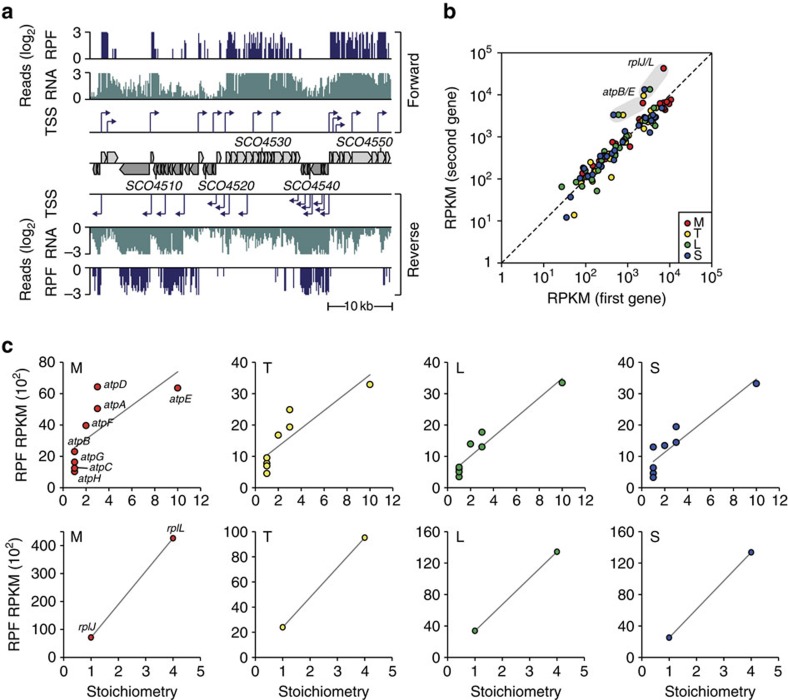
Determination of the translatome of *S. coelicolor*. (**a**) An example of visualization of transcription start sites (TSS), mRNA expression profiles (RNA) and ribosome-protected fragment profiles (RPF) at genomic region between 4,924,959 and 4,969,730. (**b**) RPF read data for the first and the second gene in the operon were compared at four growth phases: M, mid-exponential phase; T, transition phase; L, late exponential phase; S, stationary phase. (**c**) RPF data for ribosomal proteins RplJ and RplL (stoichiometry=1:4) and ATP synthase operon encoding AtpB, AtpE, AtpF, AtpH, AtpA, AtpG, AtpD and AtpC (stoichiometry=1:10:2:1:3:1:3:1) show proportional relationships with their subunit stoichiometry.

To evaluate the correlation between transcription and translation, we determined the translatome of *S. coelicolor* using the ribosome profiling (Ribo-seq) method (Supplementary Fig. 16) (ref. 24). This method enables the monitoring of protein synthesis efficiency (rates) at a genome-wide scale by using deep sequencing of ribosome-protected mRNA fragments (RPFs)^8^,^29^. To capture translating ribosomes with mRNA molecules, the mycelia at different growth phases were treated with the antibiotic thiostrepton, an inhibitor of translation elongation, followed by rapidly freezing the mycelia in liquid nitrogen, and isolation of the polysome fraction using sucrose cushions. Sequencing resulted in more than four million uniquely mapped reads with an average read length of 31 bp (Supplementary Data 7 and Supplementary Fig. 17). The RPF data were then compared directly with the transcriptome data obtained from the cultures sampled at the four different time points for in R5− liquid medium (an example is given in Fig. 4a). In this way, we could accurately discriminate protein-coding RNAs from the non-coding sRNAs (Supplementary Fig. 18); this led to the important discovery that 31 of the 230 potential sRNAs were in fact occupied by ribosomes (Supplementary Data 3), whereas the majority likely represent *bona fide* sRNAs. Longer putative sRNAs (median length=622 nt) showed higher occupancy by ribosomes than the shorter transcripts (median length=290 nt; Wilcoxon rank-sum test *P*=1.1 × 10^−11^; Supplementary Fig. 19). Eleven of the ‘coding' sRNAs (36%) are predicted to encode proteins with sequence identity to hypothetical proteins and ATP-binding proteins of other *Streptomyces* species (*e*-values<10^−5^; Supplementary Data 8) (ref. 30).

Next, we examined the proportional relationship between subunit stoichiometry in known protein complexes and their respective RPF levels. The balanced cellular concentration of each subunit of a protein complex is achieved at the translational level, rather than the transcriptional level^29^. According to this observation, we focused on the top 80 highly expressed subunits of 26 protein complexes by using the median reads per kilobase of transcript per million reads (RPKM) value of mid-exponential growth phase as a cutoff value (9.8; Supplementary Data 9). The levels of RPFs of transcripts corresponding to the first and second subunits of protein complexes were shown to be linearly correlated, indicating that the proportional relationship between subunits in protein complexes is strictly controlled at the level of translation (Fig. 4b). Furthermore, the RPF reads from mRNAs encoding non-equimolar subunits such as AtpB/E and RplJ/L were proportional to their respective subunit stoichiometries while in contrast the mRNA levels were comparable across the respective operons (Fig. 4c and Supplementary Fig. 20).

**Figure 5:**
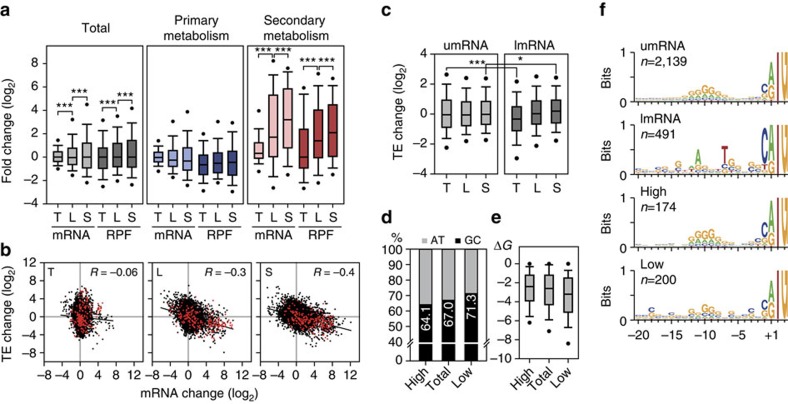
Translational buffering revealed by comparison of changes in mRNA and ribosome-protected fragment abundances. (**a**) Distribution of mRNA fold-change and RPF fold-change of total genes, primary metabolic genes and secondary metabolic genes; ***P<0.001 (Wilcoxon signed-rank test); T, fold-change between mid-exponential and transition phases; L, fold-change between mid- and late exponential phases; S, fold-change between mid-exponential and stationary phases. (**b**) Negative correlation between changes in mRNA levels and translational efficiency (TE) becomes higher at later growth phases. Red dots indicate secondary metabolic genes. (**c**) TE change distributions of umRNAs and lmRNAs across growth phases. *P<0.05; ***P<0.001 (Wilcoxon rank-sum test). (**d**) G+C content of translation initiation regions (TIR: 20 nt sequence upstream of start codon); high, genes with high TE (upper 20%); total, all coding sequences; low, genes with low TE (lower 20%). (**e**) Correlation between free energy of TIR and TE. (**f**) Conserved ribosome-binding sequences for umRNAs were observed at 8–12 bp upstream region of start codon; lmRNAs, 5′-UTR length=0. TIR of genes with high TE (High) shows more conserved polypurine (G>A) motif than genes with low TE (Low).

A systems-level comparison was undertaken between changes in levels of ssRNA-seq and Ribo-seq reads across the growth phases (Fig. 5a and Supplementary Fig. 21). We obtained global correlation coefficients between the mRNA and RPF levels of 0.85, 0.79, 0.85 and 0.89 at mid-exponential, transition, late exponential and stationary growth phases, respectively. The linear proportionality is likely to be maintained across the growth phases. Regardless of growth phase, the distribution patterns of total RNA were similar to the RPF distributions, with convergence of median values to zero. This general observation could be explained by the fact that the cellular availability of RNAP and ribosome complexes are limited, and therefore directly influences the cellular economics^29^,^31^. However, although the changes in the mRNA and RPF levels of primary metabolic genes showed similar patterns, those for secondary metabolic genes increased gradually across the growth phases. Interestingly, although the distribution of mRNA abundance widened at later growth phases, the distribution of RPF abundance retained a similar range at each growth phase; the same pattern was observed with genes of primary metabolism. This phenomenon can be explained by ‘translational buffering', where ribosome occupancy is more consistently maintained than transcript abundance^32^.

Translation efficiency (TE)—the efficiency with which an mRNA is translated—was calculated by dividing RPF levels by the corresponding mRNA transcript levels for each gene. The correlation between mRNA change and TE change provided a clear demonstration of translational buffering, evidenced by the observed negative correlation between mRNA changes and TE changes, which become more pronounced as the growth progresses towards stationary phase (Fig. 5b). Notably, secondary metabolism-related transcripts showed a tendency of increasing mRNA levels across time while the TE decreased. A striking finding is that the TE change of many secondary metabolic genes is markedly enhanced at the transition from mid-exponential to transition phase, whereas their transcription is *not* correspondingly enhanced.

We observed that the TE of lmRNAs slightly increased relative to the growth phase, whereas the TE of umRNAs did not change (Fig. 5c). With umRNAs, the change in TE is likely to converge to zero by different positive and negatively acting factors, whereas lmRNAs are likely to be controlled by common factors *en bloc*; the latter was also observed for translation of lmRNAs at stationary phase in *Escherichia coli*^33^. To investigate the correlation between the 5′-UTR and TE in relation to predicted secondary structure within the UTR, we focused on a total of 174 (top 20%) and 200 (bottom 20%) genes on the basis of TE at each growth phase. The G+C content of the 20-bp sequence upstream from the start codon was measured, revealing that the low TE group had a higher G+C content (71.3%) than the high TE group (64.1%; Fig. 5d). Similarly, umRNAs with 5′-UTRs of lower free energy tend to have a low TE (Fig. 5e). This suggests that the highly structured 5′-UTRs, along with features such as G+C content and low free energy, are likely to decrease the rate of translation of mRNAs^34^,^35^. A more conserved polypurine (G>A) motif 8–12 bp upstream region from the start codon was observed in genes from the high TE group relative to the low TE group (Fig. 5f). This motif was comparable to the general bacterial Shine-Dalgarno sequence (or RBS) and suggests that a highly conserved RBS sequence is correlated with enhanced TE in *S. coelicolor*. Taken together, these findings suggest that antibiotic productivity could be potentially improved by engineering the 5′-UTR sequence of genes involved in secondary metabolism.

### Translational buffering during secondary metabolism

**Figure 6:**
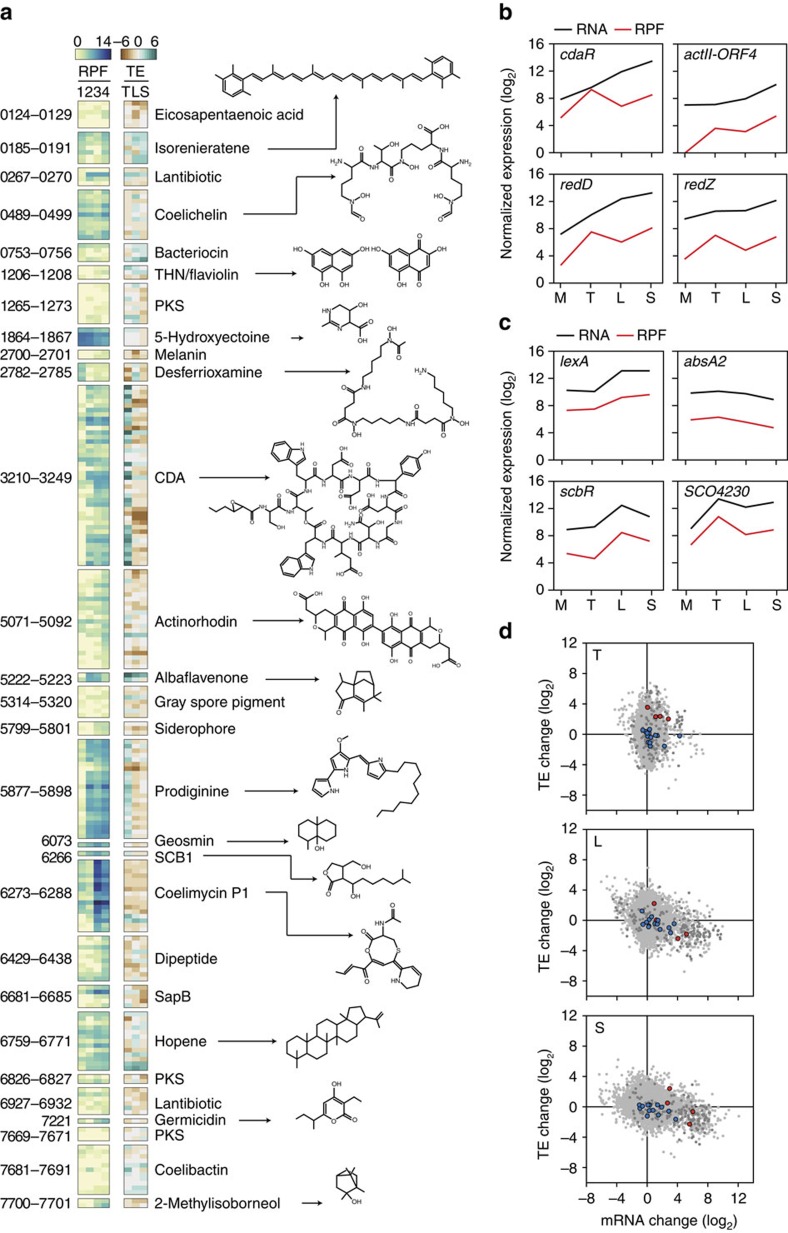
Translational landscape of secondary metabolic genes. (**a**) Ribosome-protected fragment (RPF) levels and translation efficiency (TE) fold-changes of the 221 known secondary metabolic genes in *S. coelicolor*. Leftmost numbers are the SCO gene numbers for each gene cluster. The respective known chemical structures are indicated for each secondary metabolite structure. 1, Mid-exponential phase; 2, transition phase; 3, late exponential phase; 4, stationary phase; T, fold-change between mid-exponential and transition phases; L, fold-change between mid- and late exponential phases; S, fold-change between mid-exponential and stationary phases. (**b**) mRNA and RPF levels of cluster-situated regulator-encoding transcripts for SCO3217 (CdaR), SCO5085 (ActΙΙ-ORF4), SCO5877 (RedD) and SCO5881 (RedZ) across the four growth phases. (**c**) mRNA and RPF levels of transcripts for SCO5803 (LexA), SCO3226 (AbsA2), SCO6265 (ScbR) and SCO4230, which represent other regulators of secondary metabolism. (**d**) Changes in mRNA level and TE. Red dots indicate genes encoding the cluster-situated regulators CdaR, ActΙΙ-ORF4, RedD and RedZ. Blue dots indicate other known regulators of secondary metabolism: SCO2792 (AdpA), SCO5803 (LexA), SCO3226 (AbsA2), SCO5231, SCO3063, SCO4907 (AfsQ1), SCO5260 (AtrA), SCO6008, SCO4159 (GlnR), SCO0310, SCO3932, SCO5405, SCO6265 (ScbR) and SCO4230 (PhoP). Grey dots and dark grey dots indicate, respectively, all genes and secondary metabolic genes. T, fold-change between mid-exponential and transition phases; L, fold-change between mid- and late exponential phases; S, fold-change between mid-exponential and stationary phases.

Changes in RPF patterns and TEs across the four growth phases for all genes of secondary metabolism of *S. coelicolor*^36^ are illustrated in Fig. 6a. Among 28 secondary metabolites, 4 representative antibiotics of *S. coelicolor* (CDA, actinorhodin, prodiginine and coelimycin) showed dynamic expression patterns contingent on the growth phases. However, the dynamics differed between the respective gene clusters; for example, the TE of the prodiginine and CDA clusters increased specifically from mid-exponential to transition growth phases, whereas the coelimycin and actinorhodin clusters showed maximum expression at the late exponential and stationary growth phase, respectively. Interestingly, the level of translation of the respective cluster-situated regulator (CSR) genes, *cdaR* (SCO3217), *actΙΙ-ORF4* (SCO5085), *redD* (SCO5877) and *redZ* (SCO5881), was higher at the transition growth phase than the late exponential growth phase, whereas their respective transcript levels gradually increased across the growth phases (Fig. 6b). These contrasting dynamics of transcription and translation were not observed in other regulatory genes related to secondary metabolism (Fig. 6c). Importantly, the CSR genes demonstrated highest TE changes between the mid-exponential and transition growth phases compared with other regulators of secondary metabolism, which show near zero TE changes (Fig. 6d). This unprecedented finding suggests that translational (more so than transcriptional) induction of the CSR genes is mediated specifically at the transition phase, immediately before onset of secondary metabolism.

To further investigate the growth phase-dependence of transcription and translation, we correlated the abundance of mRNA transcripts and RPFs. First, the genes were divided into 16 groups according to mRNA and RPF expression patterns, respectively, by hierarchical clustering (using Pearson correlation) and the 16 groups were analysed in a combinatorial manner, generating 256 groups (Supplementary Fig. 22a). Interestingly, some genes showed an anti-correlation between mRNA transcript and RPF levels: a group with mRNA-increasing and RPF-decreasing (ID) pattern comprised gene functions related with transport, cell membrane and macromolecule degradation (Supplementary Data 10 and Supplementary Fig. 22b), whereas another (the DI group) showed a strikingly anti-correlated trend with decreasing levels of mRNA abundance and increasing trend in RPF abundance; these include regulatory functions such as RNAP core enzyme binding, regulation (including anti-σ factors) and protein kinases (Supplementary Data 10 and Supplementary Fig. 22b). Although further studies are needed, we predict that the genes in the DI group are controlled at the translational level.

## Discussion

Systematic integration of multiple genome-wide data sets allowed us to visualize in unprecedented depth the expression of the *S. coelicolor* genome and to elucidate the relationship between transcription and translation at a genome-wide scale across four representative growth phases of this model antibiotic-producing bacterium. A total of 3,570 TSSs were identified in this study, and revealed the existence of a high proportion (21%) of leaderless transcripts; it has been speculated that this may be correlated with their capacity for antibiotic production because many antibiotics target the translation system^37^. We observed a general reduction in the translation rate of secondary metabolic genes after the transition growth phase. The results from this study suggest that translational control of gene expression is widespread in *S. coelicolor* and that it clearly influences expression of secondary metabolic genes and their associated transcription factors. The comprehensive transcriptional and translational data reported here will provide an important reference resource for molecular genetic and systems-level studies of streptomycetes. The study has also identified key genes that are subject to translational control and therefore offers target genes for investigating the molecular basis of such control. Engineering of such translational control systems offers a potentially novel route for strain engineering to enhance secondary metabolite production. Our observation that some CSRs are translationally induced at transition phase suggests one approach to synthetically manipulate secondary metabolic gene clusters.

## Methods

### Strains and cell growth

A 20% glycerol stock of *S. coelicolor* A3(2) M145 (ATCC BAA-471) spores was used to inoculate R5− liquid complex medium containing 0.16 g ml^−1^ glass beads (3 mm (±0.3 mm) diameter) and cultured to mid-exponential phase (OD_450 nm_∼0.6). Composition of the R5− liquid complex medium was as follows: 25 mM TES (pH 7.2), 103 g l^−1^ sucrose, 1% glucose, 5 g l^−1^ yeast extract, 10.12 g l^−1^ MgCl_2_·6H_2_O, 0.25 g l^−1^ K_2_SO_4_, 0.1 g l^−1^ casamino acids, 0.08 mg l^−1^ ZnCl_2_, 0.4 mg l^−1^ FeCl_3_·6H_2_O, 0.02 mg l^−1^ CuCl_2_·2H_2_O, 0.02 mg l^−1^ MnCl_2_·4H_2_O, 0.02 mg l^−1^ Na_2_B_4_O_7_·10H_2_O and 0.02 mg l^−1^ (NH_4_)_6_Mo_7_O_24_·4H_2_O. The mycelium was then diluted 1:100 into fresh R5− liquid complex medium and cultivated at 30 °C to appropriate cell density in an orbital shaker. For directional ssRNA-seq and Ribo-seq experiments, mycelium was harvested in mid-exponential phase (14 h), transition phase (18 h), late exponential phase (22 h) and stationary phase (36 h). To prepare the Ribo-seq samples, thiostrepton (Sigma), a translation elongation inhibitor, was added to cultures to a final concentration of 20 μM and subsequently incubated for 5 min at 30 **°**C before harvesting. For dRNA-seq, mycelia were cultivated under 44 different conditions as follows: four time points in the R5− liquid cultures, three time points from SMMS solid cultures, mycelia grown with 32 different nutrient combinations (10 g l^−1^ each carbon source: glucose, *N*-acetylglucosamine, glycerol and maltose; 0.5 g l^−1^ each nitrogen source: ammonium, asparagine, glutamine, serine, leucine, histidine, phenylalanine and casamino acids) in liquid minimal medium, composed of 0.5 g l^−1^ K_2_HPO_4_, 0.2 g l^−1^ MgSO_4_·7H_2_O and 0.01 g l^−1^ FeSO_4_·7H_2_O, and mycelium exposed to five different stress conditions (0.5 M sodium chloride, 1% ethanol, 42 °C heat shock, 12 °C cold shock and 0.01% SDS) for 1 h in the liquid minimal medium with 10 g l^−1^ glucose and 0.5 g l^−1^ asparagine. Cell growth was monitored by measuring the OD_450 nm_.

### RNA purification

The mycelium was resuspended in 500 μl lysis buffer composed of 20 mM Tris-HCl (pH 7.4), 140 mM NaCl, 5 mM MgCl_2_ and 1% Triton X-100. Resuspended cells were dripped into liquid nitrogen and then ground with pestle and mortar. The powdered mycelia were thawed and the cell debris was removed by centrifugation at 4 °C for 5 min at 3,000*g*. The supernatant was further clarified by centrifugation at 16,000*g* for 10 min. For ssRNA-seq and dRNA-seq samples, total RNA was isolated using miRNeasy Mini kit (Qiagen) in accordance with the manufacturer's instructions.

### ssRNA-seq library preparation

To remove genomic DNA, the isolated RNA was incubated at 37 °C for 1 h with 4 U of rDNase Ι (Ambion) and 5 μl of 10 × DNase Ι buffer (Ambion). The DNA-free RNA was purified by phenol-chloroform extraction and ethanol precipitation. Ribosomal RNA (rRNA) was removed by using Ribo-Zero rRNA Removal Kit for Meta-bacteria (Epicentre) according to the manufacturer's instructions. rRNA-depleted RNAs were checked for quality control with Agilent 2200 TapeStation system (Agilent Technologies). 200 ng mRNA was then fragmented by incubation at 70 **°**C for 5 min with 10 × Fragmentation buffer (Ambion). The reaction was terminated by adding 1 μl of Stop solution (Ambion) and the fragmented mRNA was purified by ethanol precipitation. For first strand cDNA synthesis, 3 μg of Random primers (Invitrogen) were added to the fragmented mRNA and denatured by incubation at 65 **°**C for 5 min. Then, the following was added to the reaction: 2 μl of 10 × RT buffer (Invitrogen), 1 μl of 10 mM dNTP mix, 4 μl of 25 mM MgCl_2_, 2 μl of 100 mM dithiothreitol (DTT), 1 μl of SuperScript ΙΙΙ Reverse Transcriptase (200 U μl^−1^, Invitrogen) and 1 μl of RNaseOUT (40 U μl^−1^, Invitrogen). The mixture was incubated 10 min at 25 **°**C for annealing then 50 min at 50 **°**C for reverse transcription. The reaction was terminated by incubation at 85 **°**C for 5 min. Synthesized first strand cDNA was purified by using Agencourt AMPure XP beads (Beckman Coulter). The following mixture was added to the purified cDNA for second strand synthesis: 1 μl of 10 × RT buffer (Invitrogen), 0.5 μl of 25 mM MgCl_2_, 1 μl of 100 mM DTT, 2 μl of 10 mM mixture of each dNTP (dATP, dGTP, dCTP and dUTP), 15 μl of 5 × second-strand buffer (Invitrogen), 5 μl of *E. coli* DNA polymerase (10 U μl^−1^, Invitrogen), 1 μl of *E. coli* DNA ligase (10 U μl^−1^, Invitrogen) and 1 μl of *E. coli* RNase H (2 U μl^−1^, Invitrogen). The mixture was incubated at 16 **°**C for 2 h and synthesized cDNA was purified using Agencourt AMPure XP beads (Beckman Coulter). The libraries for Illumina sequencing were constructed using TruSeq DNA Sample Prep Kit (Illumina Inc) according to the manufacturer's instructions. Briefly, the synthesized cDNA was end-repaired and 3′-ends of the blunt fragments were adenylated for the adapter ligation. The adenylated DNA fragments were ligated with Illumina adapters. A fraction of the adapter-ligated DNA between 180 and 380 bp was size-selected from a 2% agarose gel after electrophoresis. Size-selected DNA was purified by using MinElute Gel Extraction Kit (Qiagen) according to the manufacturer's instructions and eluted in 1 × TE buffer with low EDTA (10 mM Tris-HCl (pH 8.0), 0.1 mM EDTA) for the following enzyme reaction. For degradation of the second strand that contains dUTP instead of dTTP, 1 U of USER enzyme (NEB) was added to the purified DNA and incubated at 37 °C for 15 min. After 5 min incubation at 95 °C for enzyme inactivation, the library was enriched by PCR. The amplification was monitored on a CFX96 Real-Time PCR Detection System (Bio-Rad) and stopped at the beginning of the saturation point. The amplified library was purified by using Agencourt AMPure XP beads and quantified using a Qubit 2.0 fluorometer (Invitrogen).

### dRNA-seq library preparation

RNA samples from various growth conditions described previously were pooled to make 10 μg of total RNA. Genomic DNA was removed by using DNA-free Kit (Ambion) in accordance with the manufacturer's instructions. To enrich mRNA from the isolated total RNA samples, rRNA was removed by using Ribo-Zero rRNA Removal Kit for Meta-bacteria. rRNA-depleted RNA samples were verified for quality control with the Experion system (Bio-Rad). Total mRNA was split into two samples for two different libraries: the library of the primary transcriptome and the library of whole transcriptome, respectively. One unit of Terminator 5′-Phosphate-Dependent Exonuclease (TEX, Epicentre) was used to treat one of the samples to enrich primary transcripts, which have triphosphate at their 5′-ends, resulting in the preparation of the TEX-treated (TEX+) and non-treated (TEX−) samples. 2 μl of 10 × Terminator Reaction Buffer A (Epicentre) and 0.5 μl of RNaseOUT (40 U μl^−1^) were added to the TEX+ sample and incubated at 30 °C for 1 h. The reaction was terminated by adding 1 μl of 100 mM EDTA (pH 8.0). To ligate 5′-RNA adaptor, the triphosphates at the 5′-ends of mRNA were converted to monophosphate by treating with 20 U of RNA 5′-polyphosphatase (Epicentre) in a 20-μl volume containing 2 μl of 10 × RNA 5′-polyphosphatase Reaction buffer (Epicentre) and 0.5 μl of RNaseOUT (40 U μl^−1^) at 37 °C for 1 h. The mRNA was then purified by phenol-chloroform extraction and ethanol precipitation. 5 μM of 5′-RNA adaptor (5′-GUUCAGAGUUCUACAGUCCGACGAUC-3′) was added to the purified mRNA with 4 μl of T4 RNA Ligase (5 U μl^−1^, Epicentre), 2 μl of 10 × T4 RNA Ligase buffer (Epicentre), 2 μl of 10 mM ATP and 0.5 μl of RNaseOUT (40 U μl^−1^). The ligation reaction was incubated at 37 °C for 3 h. Following this step, cDNA was synthesized from adaptor-ligated RNA using random 3′ overhanging primer (N9; 5′-GTGACTGGAGTTCAGACGTGTGCTCTTCCGATCTNNNNNNNNN-3′). The primer-RNA mixture was incubated at 70 °C for 10 min then at 25 °C for 10 min. The following components were added to the reaction: 6 μl of 10 × RT buffer, 6 μl of 100 mM DTT, 3 μl of 10 mM dNTP mix, 1 μl of actinomycin D (1 mg ml^−1^), 0.75 μl of RNaseOUT (40 U μl^−1^) and 3 μl of SuperScript ΙΙΙ Reverse Transcriptase (200 U μl^−1^). The mixture was incubated 10 min at 25 °C, 1 h at 37 °C, 1 h at 42 °C and 15 min at 70 °C, sequentially. The reaction was then chilled to 4 °C. To remove residual RNAs, the reverse transcribed product was incubated at 65 °C for 30 min with 20 μl of 1 N NaOH followed by 20 μl of 1 N HCl for neutralization. Synthesized cDNA was purified using QIAquick PCR Purification Kit (Qiagen) according to the manufacturer's instructions. The cDNA was purified again by ethanol precipitation. Purified cDNA was selected at a size range between 100 and 350 bp on a 2% agarose gel by Pippin Prep (Sage Science). Size selected DNA was purified by ethanol precipitation. The purified sequencing library was then amplified by PCR with indexed primers for the Illumina sequencing platform. The success of the amplification step was monitored on a CFX96 Real-Time PCR Detection System and stopped at the beginning of the saturation point. The enriched library was then purified by ethanol precipitation. Purified libraries at a size range between 150 and 400 bp were extracted from a 2% agarose gel by Pippin Prep. The size-selected library was purified by ethanol precipitation. A second PCR amplification was carried out with a few PCR cycles to produce enough DNA for Illumina sequencing. The final amplified library was purified by ethanol precipitation and the libraries in the range 150–400 bp were extracted from a 2% agarose gel after electrophoresis. The final library was then purified using MinElute Gel Extraction Kit and quantified using Qubit 2.0 fluorometer. High-throughput sequencing is described after the ribosome profiling protocol. Approximately 92% of sequence reads (∼4.2 million sequence reads) were uniquely mapped to the *S. coelicolor* genome (NC_003888) with an average read length of 118 nt corresponding to ∼53-fold genomic coverage.

### Ribosome profiling (Ribo-seq) library preparation

The mycelium was washed in 500 μl polysome buffer composed of 20 mM Tris-HCl (pH 7.4), 140 mM NaCl, 5 mM MgCl_2_ and 20 μM thiostrepton. The cells were then resuspended in the lysis buffer (20 mM Tris-HCl (pH 7.4), 140 mM NaCl, 5 mM MgCl_2_ and 1% Triton X-100) with 20 μM thiostrepton, dripped into liquid nitrogen and then ground with pestle and mortar. The powdered cells were thawed and centrifuged at 4 °C for 5 min at 3,000*g* to remove cell debris. The supernatant was recovered and clarified by centrifugation at 16,000*g* for 10 min. To digest RNA, 750 U of RNase Ι (Ambion) was added to the cell lysate containing 50 μg total RNA in 300 μl of 10 mM Tris-HCl (pH7.6). The samples were incubated at 37 °C for 45 min with gentle rotation, followed by addition of 200 U of SUPERase·In (Invitrogen). The digested samples were carefully pipetted onto 900 μl of 1 M sucrose cushion (34% (w/v) sucrose) in a polycarbonate ultracentrifuge tube (13 × 51 mm, Beckman). Ribosomes were pelleted by centrifugation with TLA100.2 rotor (Beckman) at 215,000*g* for 4 h at 4 °C, and subsequently resuspended in 700 μl of RLT buffer (Qiagen). RNA was purified by phenol-chloroform extraction and ethanol precipitation. rRNA was removed by using Ribo-Zero rRNA Removal Kit for Meta-bacteria. The ribosome-protected RNA ‘footprint' fragments were separated by electrophoresis for 65 min at 200 V using 15% polyacrylamide TBE-urea gel (Invitrogen). RNA fragments between 26 and 32 bp were size-selected and eluted in 400 μl of RNA gel extraction buffer (300 mM sodium acetate (pH 5.5), 1 mM EDTA and 0.25% (w/v) SDS). The samples were frozen for 30 min at −80 °C then incubated overnight at room temperature with gentle mixing. Size-selected fragments were purified by ethanol precipitation and dissolved in 10 μl of 10 mM Tris-HCl (pH 8.0). The dephosphorylation reaction was carried out as follows: samples were denatured for 90 s at 80 °C; after this step, the samples were equilibrated to 37 °C and incubated for 1 h at 37 °C with 5 μl of 10 × T4 PNK buffer (NEB), 1 μl of SUPERase·In (20 U μl^−1^) and 1 μl of T4 PNK (10 U μl^−1^, NEB). Thereafter, the enzyme inactivation was performed for 10 min at 70 °C and RNA was purified by ethanol precipitation. Dephosphorylated RNA was dissolved in 8.5 μl of 10 mM Tris-HCl (pH8.0) and 1.5 μl of Universal miRNA Cloning Linker (NEB) was added to the RNA. RNA-linker mixture was denatured at 80 °C for 90 s then cooled to room temperature. The mixture was ligated in 20 μl volume with 2 μl of 10 × T4 RNA Ligase Reaction Buffer (NEB), 6 μl of PEG 8000 (50%, w/v) and 1 μl of T4 RNA Ligase 2, truncated (200 U μl^−1^, NEB). The mixture was incubated for 2.5 h at room temperature. Linker-ligated RNA was purified by ethanol precipitation and dissolved in 10 μl of 10 mM Tris-HCl (pH 8.0). For reverse transcription, 2 μl of 1.25 μM reverse transcription primer (5′-AGATCGGAAGAGCGTCGTGTAGGGAAAGAGTGTAGATCTCGGTGGTCGC-SpC18-CACTCA-SpC18-TTCAGACGTGTGCTCTTCCGATCTATTGATGGTGCCTACAG-3′; where SpC18 indicates a hexa-ethyleneglycol spacer) was added to the RNA; the mixture was denatured at 80 °C for 2 min then placed on ice. The reverse transcription reaction was prepared as follow: 12 μl of RNA-primer mixture, 4 μl of 5 × first-strand buffer, 1 μl of 10 mM dNTPs, 1 μl of 100 mM DTT, 1 μl of SUPERase·In (20 U μl^−1^) and 1 μl of SuperScript ΙΙΙ Reverse Transcriptase (200 U μl^−1^). The mixture was incubated at 48 °C for 30 min. RNA was hydrolysed by adding 2.2 μl of 1 N NaOH and incubated at 98 °C for 20 min. cDNA was purified by ethanol precipitation and separated from the unextended primer by polyacrylamide gel electrophoresis as describe above, except using DNA gel extraction buffer (300 mM NaCl, 10 mM Tris-HCl (pH 8.0) and 1 mM EDTA) instead of RNA gel extraction buffer. Circularization reaction was prepared as follow: 15 μl of cDNA, 2 μl of CircLigase 10 × Reaction Buffer (Epicentre), 1 μl of 1 mM ATP, 1 μl of 50 mM MnCl_2_ and 1 μl of CircLigase ssDNA Ligase (100 U μl^−1^, Epicentre). The mixture was incubated at 60 °C for 1 h followed by enzyme heat-inactivation by incubation at 80 °C for 10 min. The sequencing library was amplified from the circulated DNA by PCR. PCR amplification was performed with varying numbers of cycles in order to determine the optimal cycle. The amplified library was separated from the unextended primers by electrophoresis for 40 min at 180 V using an 8% polyacrylamide gel. The final library was extracted from the gel slice as described above, and quantified using a Qubit 2.0 fluorometer.

### High-throughput sequencing

The resulting library was loaded onto a flow-cell and sequenced using an Illumina Miseq v.2 instrument. The 50-bp read recipe was used for the ssRNA-seq and Ribo-seq libraries, and the 150-bp read recipe was used for dRNA-seq libraries in accordance with the manufacturer's instructions. The ssRNA-seq generated more than 120 million reads with an average read length of 50 bp and the number of sequence reads for each library ranged from 1.0 × 10^7^ to 1.7 × 10^7^. The quality-processing steps yielded 76.5–87.6% of these reads as uniquely mapped onto the *S. coelicolor* genome, corresponding to 75.4-fold average genomic coverage (602-fold in total). The rRNA depletion method used in this study efficiently enriched the mRNA molecules for the cDNA library construction^18^,^38^.

### Data processing

The linker sequence was trimmed from reads of the Ribo-seq libraries before being aligned to the genome. Reads that were shorter than 25 bp after trimming or did not contain the linker sequence were discarded. Also, the random 3′-overhanging (N9) sequences in reads of dRNA-seq library were trimmed. Reads shorter than 25 bp after trimming were discarded. The reads were then aligned to the *S. coelicolor* genome (NC_003888, ftp://ftp.ncbi.nlm.nih.gov/genomes/Bacteria/Streptomyces_coelicolor_A3_2__uid57801/) using CLC Genomics Workbench (CLC bio) with the following parameters: mismatch cost 2, deletion cost 3, insertion cost 3, length fraction 0.9 and similarity fraction 0.9. Only uniquely mapped reads were retained. To validate the reproducibility between duplicate data, the expression of genes was normalized by RPKM. High coefficient of determination (*R*^2^≥0.96) calculated between duplicate data confirmed high reproducibility (Supplementary Fig. 8b). The expression of the genes was normalized using the DESeq package in R^39^, and the expression values for replicate data were merged as a normalized value. For all transcriptomic analysis, the count value after normalization by DESeq was used. To estimate the numbers of transcripts present at biologically relevant expression levels, we assumed that the normalized expression levels of the secondary metabolic genes (221 genes in total) are minimal at the mid exponential growth phase^40^ and therefore we used the expression levels of these genes as cutoff value (median=32.8). Among the four time points, only genes that had normalized mRNA expression level greater than 32.8 for at least one time point were considered. Comparing every two time points, genes having mRNA fold changes over 2 and *P*-value smaller than 0.05 (calculated by DESeq) were subsequently included in the analysis. 4,557 genes satisfying these criteria were hierarchically clustered (method=manhattan, complete) by using R, then visualized as a heat map in log_2_ scale. For calculation of TE, an arbitrary value of 1 was added to all data to avoid a zero value denominator. Because the sequences of the first 20 genes and the last 20 genes of *S. coelicolor* represent duplications, these 40 genes were excluded from all analysis.

### TSS identification and data analysis

Genomic positions of the 5′-ends of uniquely aligned dRNA-seq reads from the TEX-treated (TEX+) libraries were considered to be potential TSSs. TSSs were then determined as described previously, followed by manual curation^41^. Briefly, potential TSSs within 100 bp were clustered together, partitioning the 8.7 Mb *S. coelicolor* genome into 11,916 clusters; then, adjacent peaks in each cluster were sub-clustered by calculating the standard deviation of their genomic positions to select a local maximum peak as the TSS in each sub-cluster. If two or more peaks located nearby had standard deviation of <10 they were sub-clustered together. During this sub-clustering step, if the total read count in a cluster or a sub-cluster is less than three, the cluster or the sub-cluster was removed. For example, in a case where three peaks are positioned at 100, 114 and 128, only the first two peaks are grouped in the same cluster because the standard deviation of (100, 114) is 9.9, whereas the standard deviation of (100, 114, 128) is 14. On the other hand, if there are 29 peaks at every position from 100 to 128, they are grouped in one cluster because the standard deviation of the 29 genomic positions is 8.5. Thus, standard deviation measure is the criterion required to sub-cluster peaks that are densely located in a certain region of the genome. Then, the peak with maximum reads in each sub-cluster was designated as the TSS. Among the selected TSSs in one cluster, if two or more TSSs were closely located (standard deviation of genomic positions <10) the lower peaks were removed. Finally, we compared the assigned TSSs with data from the respective non-TEX-treated libraries. If the peak is not found in TEX− data within ±5 bp, the peak was removed. Further, manual curation was performed by comparing the data with the corresponding ssRNA-seq profiles. If the expression level of a gene (or operon) is extremely high, the internal peaks from processed RNAs are not properly removed by the steps described above, and therefore we manually removed those peaks. In contrast, if a peak is present in the TEX+ library and has a clear ssRNA-seq profile, this was selected as a TSS. Among the TSSs located from 500 bp upstream to 150 bp downstream of the respective annotated start codon of each open reading frames, the TSS scoring the maximum number of reads was classified as the primary (P) and the others as secondary TSSs (S). The mapped TSSs were compared with previously known TSSs (within the range of ±4 bp of our data). All data were visualized using SignalMap (Roche NimbleGen, Inc.)^16^,^18^.

### Motif discovery

The conserved promoter sequence analysis was conducted using the MEME software. We first extracted the 20-bp upstream sequences of each TSS (3,570 in total) to identify the −10 motif. After obtaining the conserved sequence (TANNNT), we extracted 8 bp upstream and 7 bp downstream sequences from the first and the last nucleotide of the conserved sequence, respectively. The 21-bp (N_8_TANNNTN_7_) sequences were then used to draw the conserved motif sequences using Weblogo^42^. Then, the sequences between 40 and 25 bp upstream of each TSS were extracted to obtain −35 motif (NTGACC). The 16-bp sequences composed of 4 bp upstream and 6 bp downstream sequences (N_4_NTGACCN_6_) was extracted and used to obtain the conserved sequence. Then, the variable length of the space between −10 and −35 was calculated for each promoter. To obtain the −10 and −35 motifs for promoters associated with each class of transcript—lmRNAs, primary TSSs, antisense TSSs, internal TSSs, intergenic TSSs and sRNAs—we repeated the procedure described above. A conserved hexanucleotide −10 motif (that is, TANNNT) was found from 80.4% (2,870 out of 3,570; *P*<0.05; MEME) of the identified TSSs, which is similar to the recently reported −10 motif for *Mycobacterium tuberculosis*^25^. This is consistent with the observation that the number of conserved nucleotides within the −10 motif tends to diminish when genomic G+C content is above 60% (ref. 43). It is worth noting that the average G+C content of the *S. coelicolor* promoter region is 63.9%, contrasting with that of the *S. coelicolor* genome as a whole of 72.1%. Sequences and length of spacer between the 5′ end of the −10 hexamer and the 3′-end of the −35 hexamer have been shown to have significant influence on bacterial promoter activity because this region is specifically recognized by each σ-factor. To test this, the promoters with identified −10 and −35 consensus regions were grouped on the basis of spacer length, resulting in 62.1% (1,036 out of 1,667) of them having the spacers between 15 and 20 nt (Supplementary Fig. 5).

### 5′-Rapid amplification of cDNA ends

Specific primers were designed to amplify 200–300 bp covering the downstream regions of selected TSSs. Cells were harvested in late exponential phase (22 h). The mycelium was resuspended in 500 μl lysis buffer composed of 20 mM Tris-HCl (pH 7.4), 140 mM NaCl, 5 mM MgCl_2_ and 1% Triton X-100. Resuspended cells were dripped into liquid nitrogen and then ground with pestle and mortar. The powdered mycelia were thawed and the cell debris was removed by centrifugation at 4 °C for 5 min at 3,000*g*. The supernatant was further clarified by centrifugation at 16,000*g* for 10 min. Total RNA was isolated using miRNeasy Mini kit (Qiagen) in accordance with the manufacturer's instructions. To remove genomic DNA, 10 μg of the isolated RNA was incubated at 37 °C for 1 h with 4 U of Turbo DNase (Thermo Fisher Scientific) and 5 μl of 10 × Turbo DNase buffer (Thermo Fisher Scientific). The DNA-free RNA was purified by phenol-chloroform extraction and ethanol precipitation. To enrich mRNA from the isolated total RNA samples, rRNA was removed by using Ribo-Zero rRNA Removal Kit for Meta-bacteria. 5′-Tag-cDNA library was constructed as described previously with optimized amounts of reagents^44^. Briefly, 50 μM of short RNA adaptor (5′-ACGGACUAGAAGAAA-3′) was added to 1 μg of the rRNA-depleted RNAs with 10 U of T4 RNA ligase (Thermo Fisher Scientific). The ligation reaction was incubated at 37 °C for 90 min, then 70 °C for 10 min. The adapter ligated RNAs were purified using Agencourt AMPure XP beads (Beckman Coulter). Half of the recovered RNAs were then treated with 20 U of RNA 5′-polyphosphatase (TAP; Epicentre). Identical treatments were applied to the other half of the RNAs, except that RNA 5′-polyphosphatase was omitted, providing a negative control. Both reactions were incubated at 37 °C for 1 h, then the RNAs were purified by ethanol precipitation. RNA 5′-polyphosphatase-treated sample (TAP+) and negative control (TAP−) were ligated to 10 μM of second RNA adapter (5′-AUAUGCGCGAAUUCCUGUAGAACGAACACUAGAAGAAA-3′) with 10 U of T4 RNA ligase (Thermo Fisher Scientific). The adapter ligated RNAs were purified using Agencourt AMPure XP beads (Beckman Coulter). cDNA was synthesized from the adapter ligated RNAs with SuperScript ΙΙΙ First-Strand Synthesis System (Invitrogen) according to the manufacturer's instruction. Synthesized cDNA was purified twice using Agencourt AMPure XP beads (Beckman Coulter). Then, the cDNA was amplified with 10 μM of the selected gene-specific primer and 25 μM of primer (5′-GCGCGAATTCCTGTAGAACG-3′), which is complementary to second RNA adapter. The sequences of gene-specific primers are indicated in Supplementary Table 3. Amplified products were separated by gel electrophoresis.

### Quantitative real-time PCR

First-strand cDNA was synthesized from 8 μg of total RNA by using SuperScript ΙΙΙ First-Strand Synthesis System (Invitrogen) in accordance with the manufacturer's instructions. The amplification of the cDNAs was monitored on a CFX96 Real-Time PCR Detection System with SYBR Green I Nucleic Acid Gel Stain (Invitrogen) under the following conditions: 98 °C for 10 s; 62 °C for 30 s; 72 °C for 10 s for 35 cycles. The sequences of primers used for amplification are indicated in Supplementary Table 4.

### Data access and in-house scripts availability

The reference genome for *S. coelicolor* A3(2) strain M145 (accession code NC_003888) is available from National Center for Biotechnology Information. In-house scripts used for data processing are available at http://cholab.or.kr/data/.

## Additional information

**Accession codes:** All raw and processed data have been deposited in the Gene Expression Omnibus archive under accession code GSE69350.

**How to cite this article:** Jeong, Y. *et al*. The dynamic transcriptional and translational landscape of the model antibiotic producer *Streptomyces coelicolor* A3(2). *Nat. Commun.* 7:11605 doi: 10.1038/ncomms11605 (2016).

## Supplementary Material

Supplementary Information Supplementary Figures 1-22, Supplementary Tables 1-4, Supplementary References

Supplementary Dataset 1 Listing of all transcription start-sites (TSSs) of *S. coelicolor* identified in this study by dRNA-seq.

Supplementary Dataset 2 Functional categorization of genes encoded by leaderless transcripts.

Supplementary Dataset 3 Catalogue of small RNAs identified in this study.

Supplementary Dataset 4 Normalized mRNA expression levels at the four growth phases examined as deduced from the ssRNA-seq data.

Supplementary Dataset 5 Compilation of genes differentially expressed at different growth phases.

Supplementary Dataset 6 Transcriptome dynamics at different growth phases.

Supplementary Dataset 7 Normalized ribosome-protected mRNA fragment (RPF) levels at four growth phases.

Supplementary Dataset 8 PSI-BLAST similarity search results for predicted peptide sequences from novel sRNA transcripts.

Supplementary Dataset 9 List of the protein subunits of known protein complexes used in stoichiometry analysis, their functions and RPF data for each growth phase.

Supplementary Dataset 10 List of the genes in II, DD, ID and DI groups, their mRNA and RPF cluster numbers and known or predicted functions.
