# Supplementary material for: The dynamic transcriptional and translational landscape of the model antibiotic producer Streptomyces coelicolor A3(2)
Source: Nat Commun. 2016 Jun 2;7:11605. doi: 10.1038/ncomms11605 (PMC4895711; doi:10.1038/ncomms11605)
Supplement: Supplementary Information — Supplementary Figures 1-22, Supplementary Tables 1-4, Supplementary References [file ncomms11605-s1.pdf]

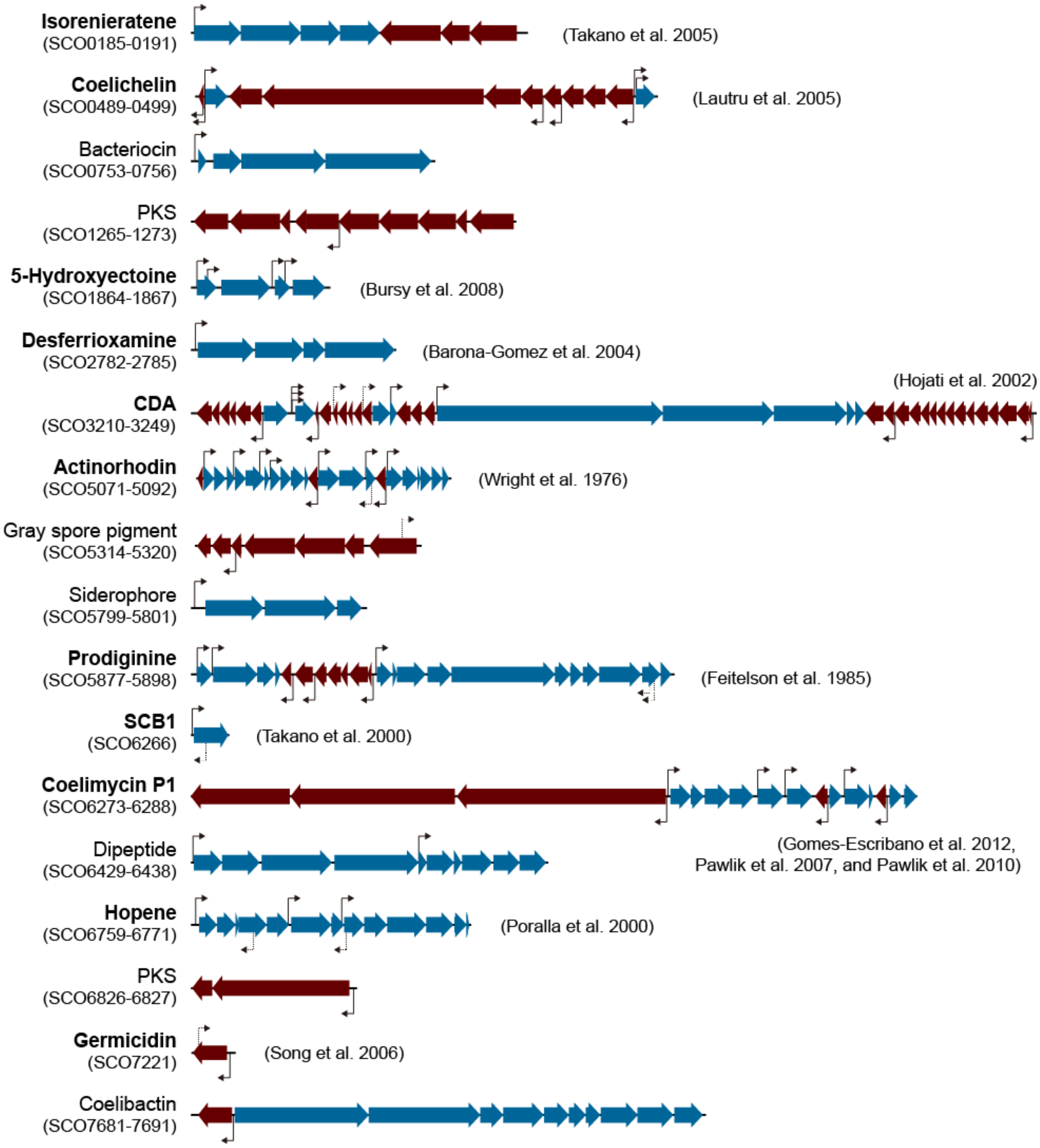

Supplementary Figure 1. TSSs assigned to the secondary metabolic gene clusters. Primary and secondary TSSs are indicated as solid lines and the others are indicated as dotted lines.

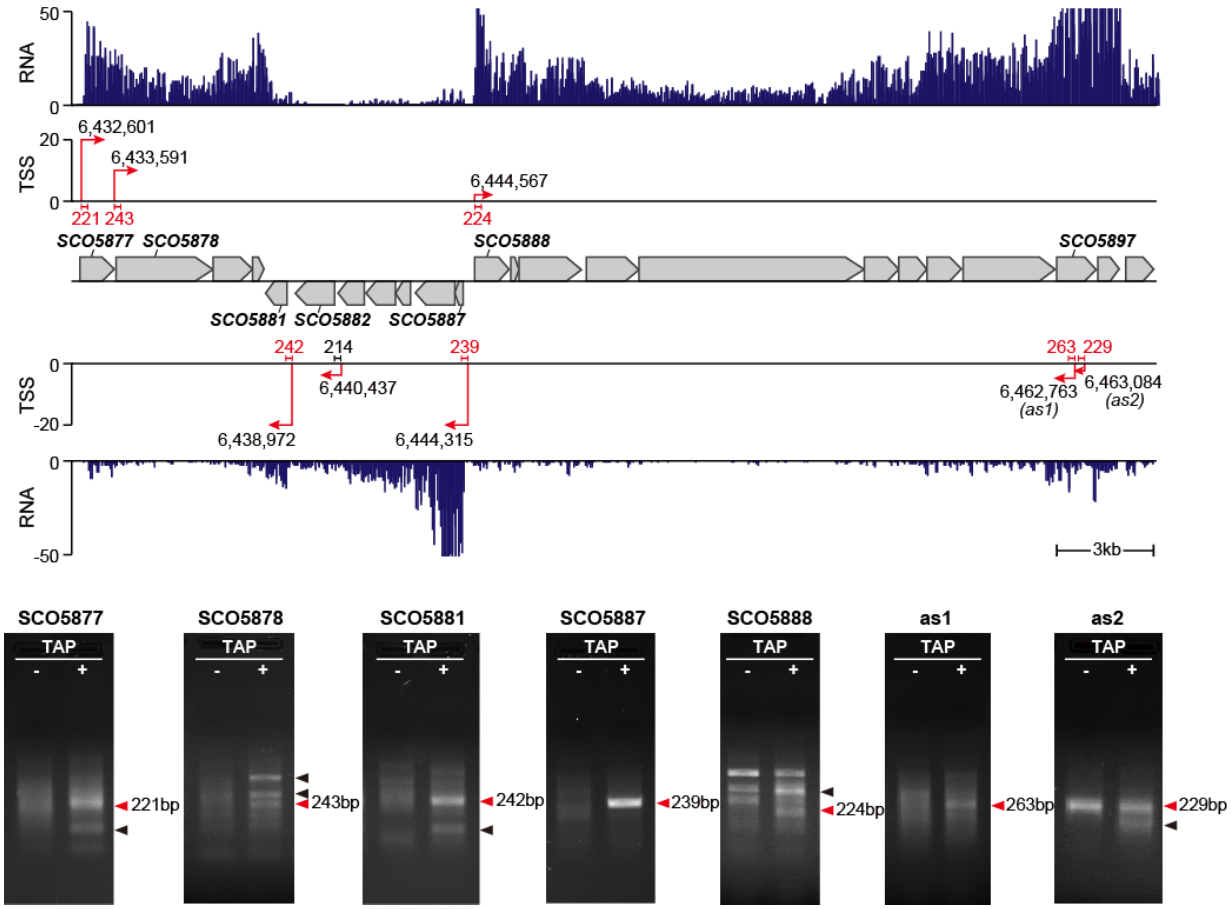

Supplementary Figure 2. Targets of 5'-RACE in prodiginine clusters and 5'-RACE results. 5'-RACE-based TSS mapping was used to confirm the identified TSSs in this study specifically for 8 TSSs (6 Primary and 2 Antisense) in the prodiginine cluster (**upper**). We designed specific primers to amplify 200 to 300 bp covering the downstream region of selected TSSs. As a result, seven out of eight TSSs showed bands of desired sizes for the prodiginine cluster (**lower**). We predicted that the reason why the TSS determined for SCO5882 was not confirmed by 5' RACE is that its expression is very low. Since the TSS prediction was done by pooling 44 different growth conditions, the TSS of SCO5882 gene might have been detected from a growth condition different from the one selected (liquid R5−). Additional bands (black arrows) indicate possible alternative start sites or unrelated primary transcripts detected by primer binding at other loci[1].

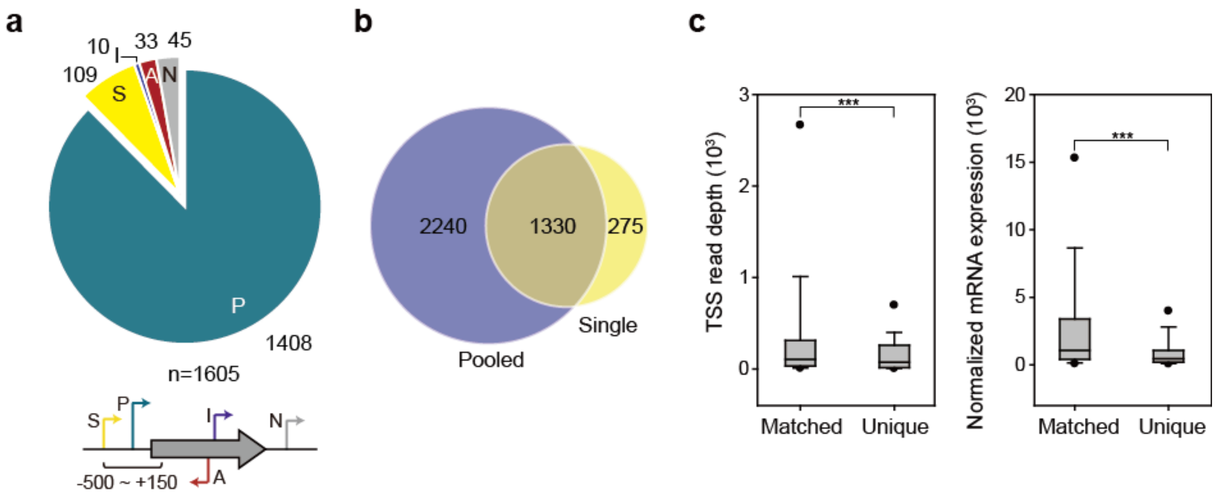

Supplementary Figure 3. Independent dRNA-seq experiment results from a single mid-exponential phase culture. We obtained 3,778,410 and 3,723,602 reads for TEX-treated (TEX+) and untreated (TEX–) samples, respectively. Among them, total mapped reads were 90% (3,418,653) for TEX+ sample and 93% (3,479,458) for TEX– sample, and uniquely mapped reads were 77% (2,923,347) for TEX+ sample and 89% (3,318,625) for TEX– sample. Average read lengths were 62.5 bp and 100.5 bp for TEX+ and TEX– samples, respectively. The data analysis, performed as that described in the **Methods**, resulted in the identification of 1,605 TSSs. (**a**) Categorization of TSSs identified in single condition sample. (**b**) Comparison between TSSs from pooled sample and single condition sample. The results overlapped in 83% of the mapped TSS within 10 bp, indicating high reproducibility between single and pooled libraries. (**c**) TSS read depth and normalized mRNA expression distribution found in both pooled and single condition samples (**Matched**) and TSSs only found in single condition sample (**Unique**). The 17% (275) TSSs that did not correlate with TSSs described in the manuscript were examined further. Among these 275 TSSs, 143 were represented only in TEX- library suggesting that the transcripts may be degraded by TEX enzyme; the remaining 132 unique TSSs that were not found in both TEX+ and TEX- samples described in the manuscript have relatively low read depths and mRNA expression levels compared to 1,330 matched TSSs. We suggest that the signals of the 132 unique TSSs were underrepresented in pooled library because of their low expression levels (potentially diluted considerably within the pool of 44 RNA samples, particularly if these TSS are specific to mid-exponential growth phase). ***, Wilcoxon rank-sum test p-value < 0.001.

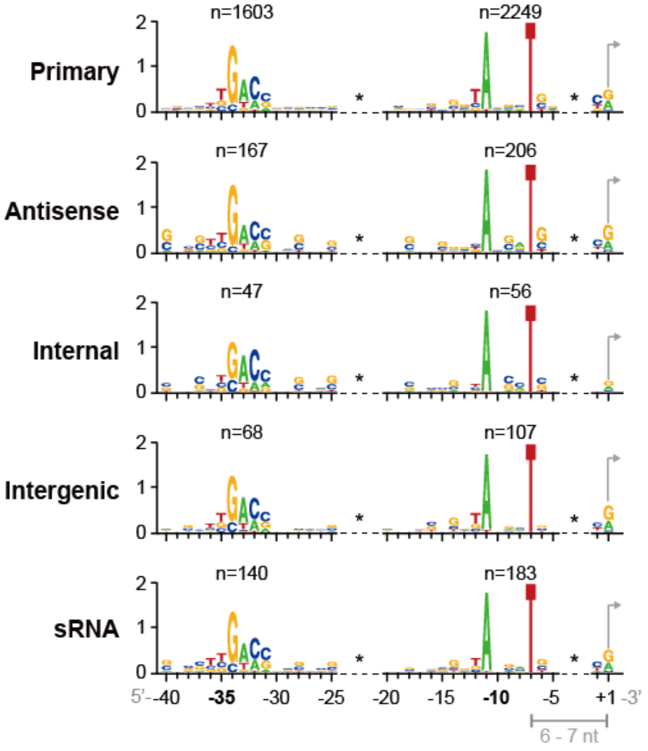

Supplementary Figure 4. Conserved −10 and −35 motifs promoter motifs observed for each class of TSS. An evaluation of the sequence conservation of promoter elements from each class of TSSs resulted in the identification of the same −10 motif from 70.9% of internal TSSs, 80.5% of antisense TSSs, and 81.7% of intergenic TSSs.

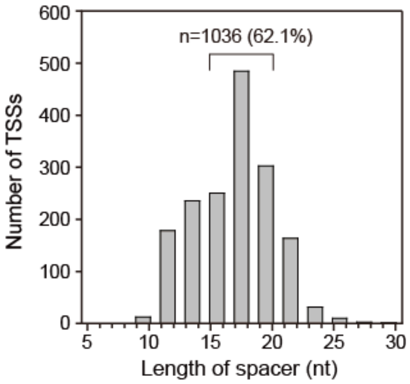

Supplementary Figure 5. Distance between the −10 and −35 motifs. The promoters with identified −10 and −35 consensus regions were grouped on the basis of spacer length, resulting in 62.1% (1,036 out of 1,667) of them having the spacers between 15 to 20 nt. The mean spacer length is 17.2 nt, which is similar to typical *E. coli* promoters recognized by housekeeping σ-factor (the optimal spacer length: 17 ± 1 nt).

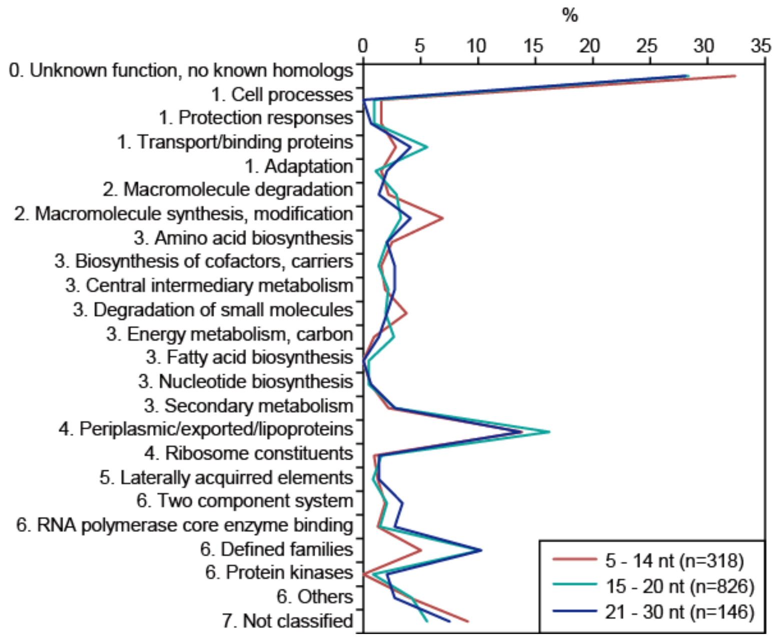

Supplementary Figure 6. Functional classification according to the distance between the −10 and −35 motifs. Genes were grouped by their length of spacers, and all three groups comprised similar functional categories; **0**, Unknown function, no known homologs; **1**, Cell processes; **2**, Macromolecule metabolism; **3**, Metabolism of small molecules; **4**, Cell envelope; **5**, Extrachromosomal; **6**, Regulation; **7**, Not classified (including putative assignments). Regardless of their lengths, all spacers are associated with genes of a variety of cellular functions including secondary metabolic functions.

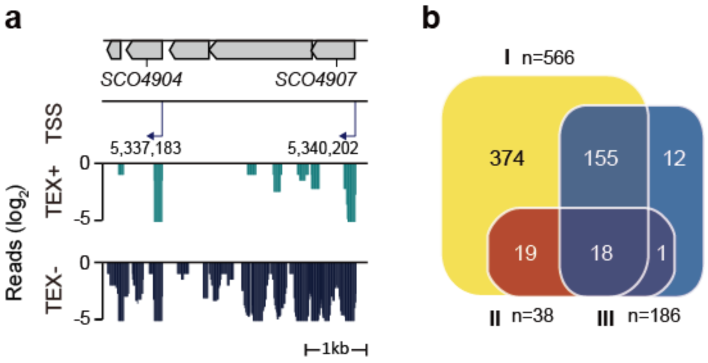

Supplementary Figure 7. Leaderless transcripts. (**a**) An example of dRNA-seq profile of lmRNAs (SCO4904 and SCO4907). (**b**) Comparison between lmRNAs identified in this study and previous studies. Most previously identified lmRNAs (192 out of 205) were among the lmRNAs reported in this study. **I**, this study; **II**, Ref.[2].; **III**, Ref.[3].

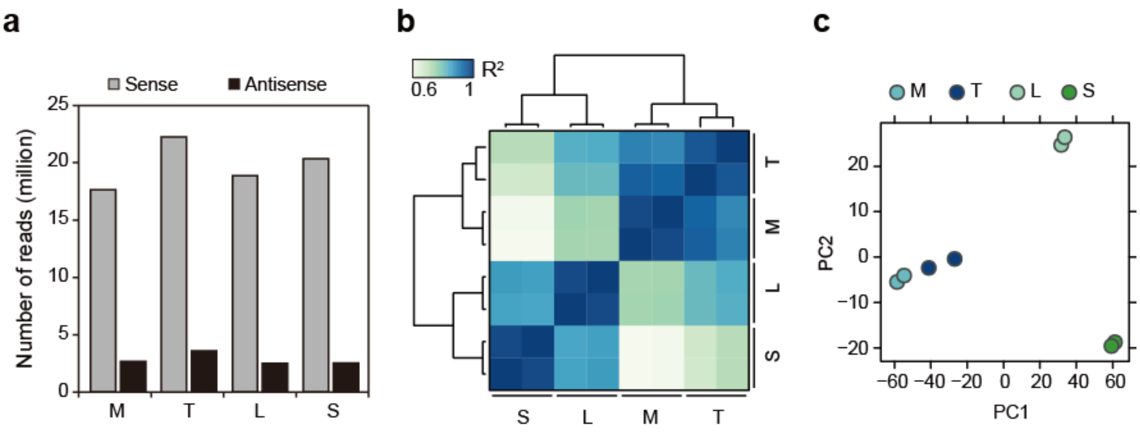

Supplementary Figure 8. Quality of ssRNA-seq data. (**a**) To test strand-specificity of the mapped reads against the current annotation, we calculated the ratio between fractions of reads mapped to the sense and antisense strands of the CDS regions. Only 14% of the reads mapped to the antisense strand, indicating that the dUTP –based method worked efficiently for *S. coelicolor*. **M**, mid exponential phase; **T**, transition phase; **L**, late exponential phase; **S**, stationary phase. (**b**) The ssRNA-seq results were highly reproducible ($R^2 \geq 0.96$). (**c**) PCA analysis indicated the ssRNA-seq data could be summarized by two principal components, accounting for almost 94% of the total variability. It resulted in four distinct clusters based on the four growth phases.

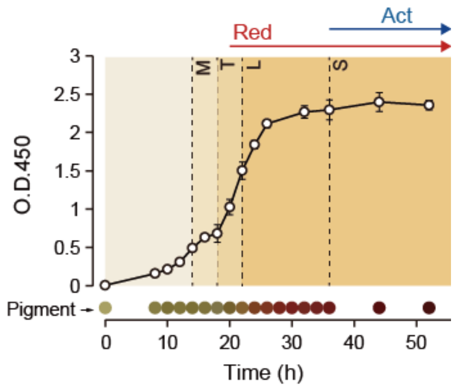

Supplementary Figure 9. Growth curve of *S. coelicolor* grown in R5− medium. RNA samples were obtained at mid-exponential (**M**), transition (**T**), late exponential (**L**), and stationary (**S**) phase. Red and blue arrows indicate the onset of production of prodiginine (Red) and actinorhodin (Act), respectively. The synthesis of pigments was represented by the changes in colour of medium.

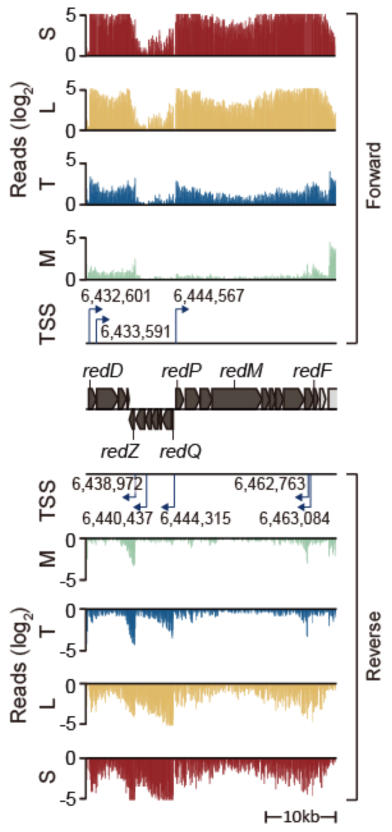

Supplementary Figure 10. Differential expression pattern of prodiginine gene cluster across different growth phases. M, mid-exponential phase; **T**, transition phase; **L**, late exponential phase; **S**, stationary phase.

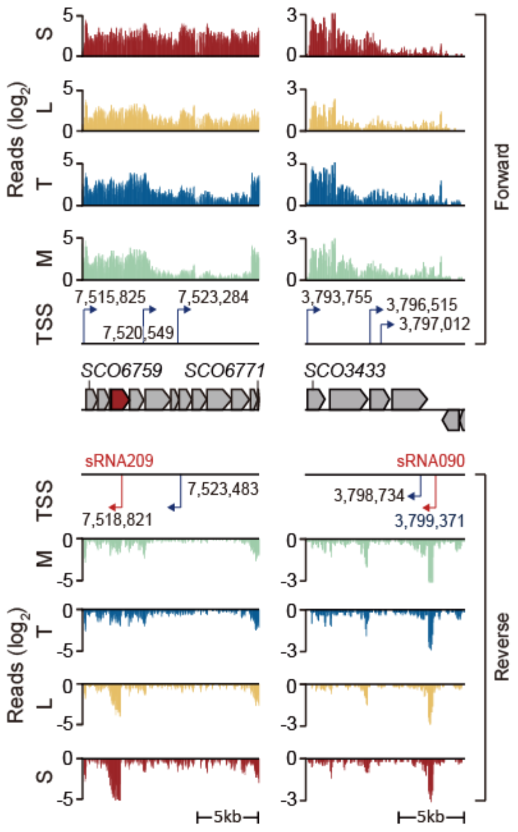

Supplementary Figure 11. Examples of potential antisense sRNAs: sRNA209 and intergenic sRNA090. Transcription of sRNA209 was significantly increased at late exponential and stationary growth phases. Interestingly, the SCO6762 in the sense strand encodes the phytoene dehydrogenase in the hopanoid biosynthetic gene cluster, which has been proposed to direct the synthesis of both hopene and the related aminotrihydroxybacteriohopane[4]. In addition, it has been suggested that sRNA209 may regulate gene expression of SCO6762 which in turn could modulate the synthesis of the hopanoid compounds[5]. However, in the gene cluster (SCO6759-SCO6771), the first five genes (SCO6759-SCO6763) showed constant expression levels across all growth phases, whereas the following eight genes (SCO6764-SCO6771) were strongly activated at the stationary growth phase. Furthermore, there are two transcription units transcribed from two TSSs (7,515,825 and 7,520,549) in front of SCO6759 and SCO6764, respectively, suggesting that sRNA209 may have a role in the regulation of the second part of the gene cluster. In contrast, sRNA090 between SCO3436 and SCO3437 showed constant expression levels across the growth phases suggesting that sRNA090 may act as a trans-acting regulatory sRNA. **M**, mid-exponential phase; **T**, transition phase; **L**, late exponential phase; **S**, stationary phase.

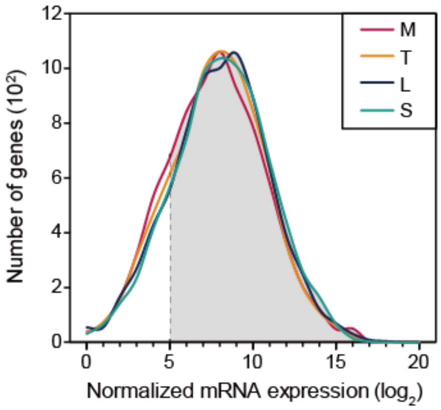

Supplementary Figure 12. Distribution of mRNA transcript levels at each growth phase. Due to the high sensitivity of the ssRNA-seq method transcripts were detected for most genes; proportion of transcribed genes having sequence reads of >2 was approximately 99%. Even though the sequence reads were filtered through the quality-processing step, we anticipate that the estimate of transcribed genes contains false positives. To estimate the numbers of transcripts present at biologically relevant expression levels, we assumed that the normalized expression levels of the secondary metabolic genes (221 genes in total) are minimal at the mid-exponential growth phase[4]. Using the expression levels of these genes as cut-off value (median = 32.8; indicated as dotted line), we scored 85% genes (6,693 in total from all four-time points) as having expression levels above the cut-off value. **M**, mid-exponential phase; **T**, transition phase; **L**, late exponential phase; **S**, stationary phase.

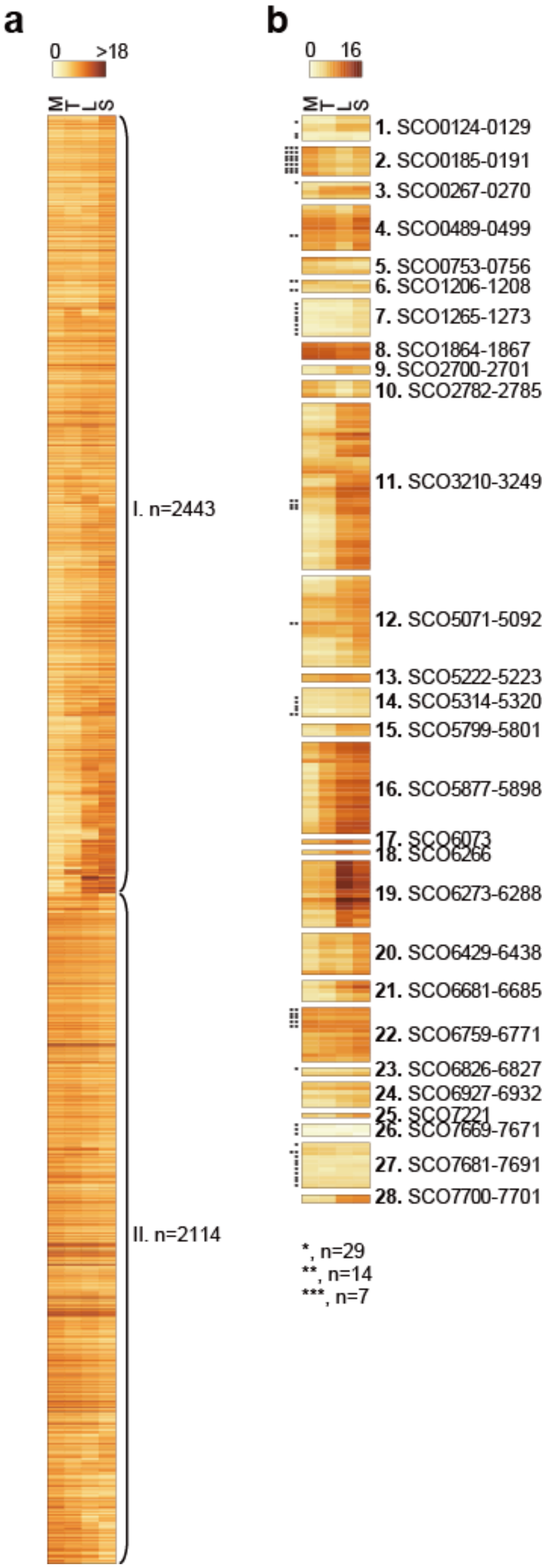

Supplementary Figure 13. Transcriptome dynamics across growth phases.
(**a**) Hierarchical clustering analysis generated two large expression clusters, in which 2,445 and 2,113 genes were induced or repressed between the growth phases. Transcription of ~1,000 genes was significantly induced or repressed at the transition growth phase whereas more than 4,000 genes were altered under the late exponential or stationary growth phases.

(**b**) Among the 221 genes in the 28 annotated secondary metabolic gene clusters of *S. coelicolor*, 171 were induced at the transition, late exponential or stationary growth phase. For example, the genes in the prodiginine (SCO5877-SCO5898) and CDA (SCO3210-SCO3249) biosynthetic pathways were mostly activated at the late exponential growth phase. In contrast, the actinorhodin biosynthetic pathway genes (SCO5071-SCO5092) were expressed later at the stationary growth phase, consistent with the appearance of the pigments.

M, mid-exponential phase; **T**, transition phase; **L**, late exponential phase; **S**, stationary phase; *, expression ≤ 32.8 reads cut-off; **, fold-change < 2; ***, p-value ≥ 0.05 (DESeq); Secondary metabolite clusters: 1, Eicosapentaenoic acid; 2, Isorenieratene; 3, Lantibiotic; 4, Coelichelin; 5, Bacteriocin; 6, THN/flaviolin; 7, PKS; 8, 5-Hydroxyectoine; 9, Melanin; 10, Desferrioxamine; 11, CDA; 12, Actinorhodin; 13, Albaflavenone; 14, Gray spore pigment; 15, Siderophore; 16, Prodiginine; 17, Geosmin; 18, SCB1; 19, Coelimycin P1; 20, Dipeptide; 21, SapB; 22, Hopene; 23, PKS; 24, Lantibiotic; 25, Germicidin; 26, PKS, 27, Coelibactin; 28, 2-Methylisoborneol.

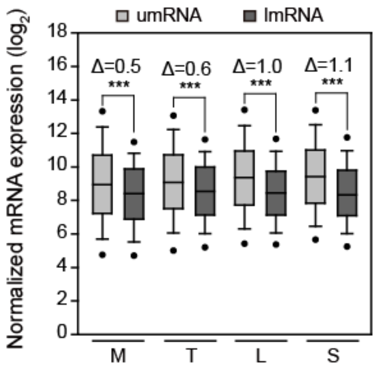

Supplementary Figure 14. Distribution of mRNA transcript levels of umRNAs and lmRNAs at each growth phase. Median value of lmRNA levels was lower than median value of umRNA expression levels; ***, $p < 0.001$ (Wilcoxon rank-sum test). The differences between median values of lmRNAs and umRNAs at each growth phase are indicated. **M**, mid-exponential phase; **T**, transition phase; **L**, late exponential phase; **S**, stationary phase.

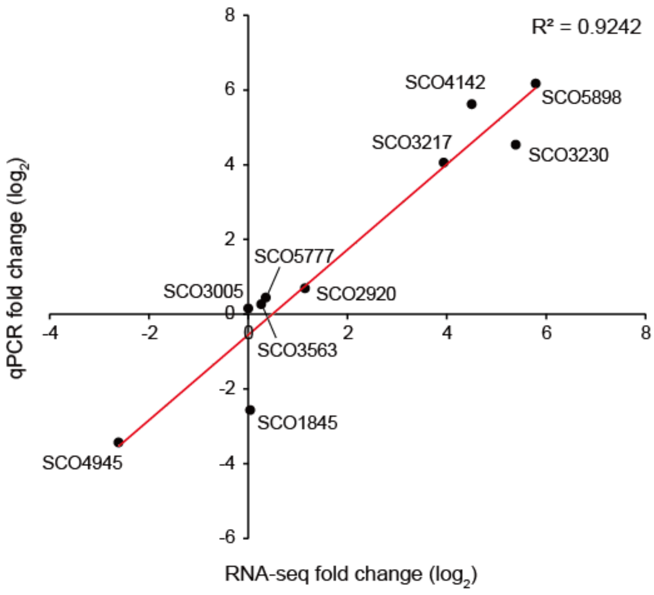

Supplementary Figure 15. **Comparison between fold-changes from ssRNA-seq and qPCR.** To show that the changes in mRNA expression levels are reproducible, we performed qPCR on a few target genes which have various fold changes between mid- and late exponential phase. qPCR was performed using RNA samples extracted from cultures grown to the same time point (mid- and late exponential phase) as described in the **Methods**. The SuperScript III reverse transcriptase (Invitrogen) was used to first synthesize cDNAs and SYBR Green I Nucleic Acid Gel Stain (Invitrogen) was used to measure the different expression level of these genes. The expression fold changes from qPCR were calculated from the Ct values. The comparison of the ssRNA-seq analysis and qPCR results showed a R^2 value of 0.92, suggesting that our results are highly genuine and reproducible.

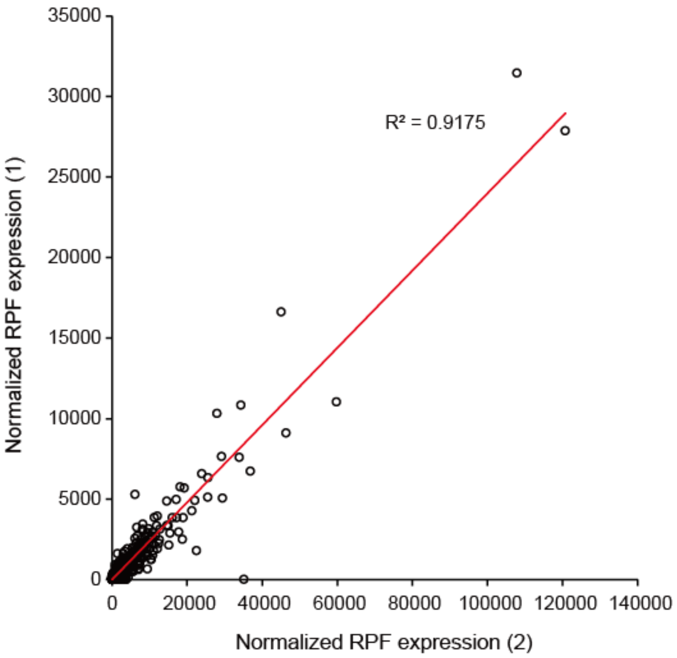

Supplementary Figure 16. Reproducibility of Ribo-seq results. To confirm the reproducibility of the Ribo-seq data, we performed another Ribo-seq experiment using RNA samples extracted from mid-exponential phase using the same method as that described in the **Methods**. The comparison of this data (2) with the previous data described in the main text (1) resulted in a coefficient of determination (R^2) value of 0.92 suggesting the results are highly reproducible.

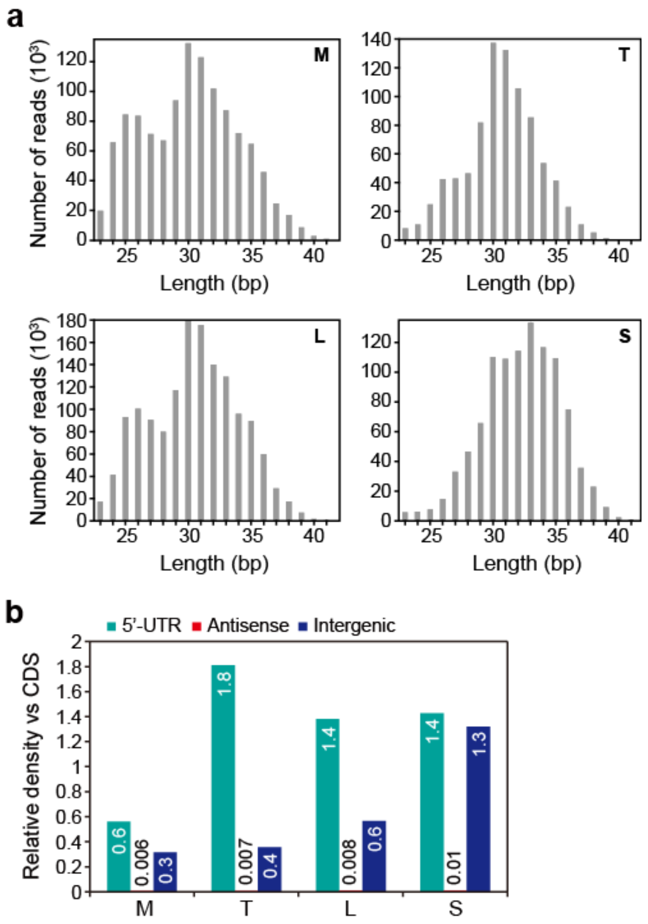

Supplementary Figure 17. Quality control results of Ribo-seq. (**a**) Read length distribution. (**b**) Density of Ribo-seq reads on 5' UTR, antisense, intergenic region relative to the CDS region. To compare the density of mapped reads between CDS, 5' UTR, antisense, and intergenic region, total mapped reads were counted for each part, and the read counts were divided by total length of each part. Then, the calculated read densities of 5' UTR, antisense, and intergenic region were divided by the read density of CDS. 5' UTR read density is highest at every growth phases except mid-exponential phase, where CDS read density is the highest. Interestingly, the read density in intergenic region markedly increased at stationary phase. **M**, mid-exponential phase; **T**, transition phase; **L**, late exponential phase; **S**, stationary phase.

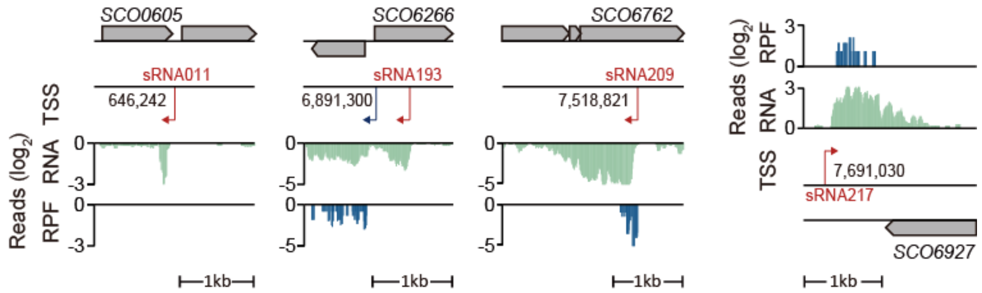

Supplementary Figure 18. Examples of potential antisense sRNAs (sRNA193 and sRNA209) and intergenic sRNAs (sRNA011 and sRNA217). Notably, sRNA209 and sRNA217 yield RPF profiles indicating that they are translated.

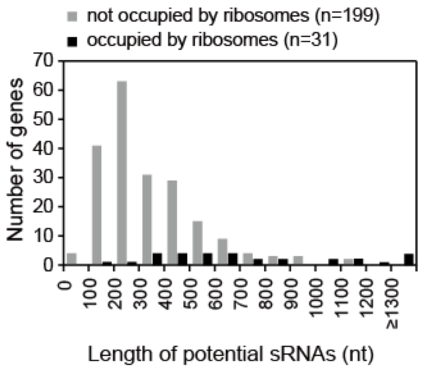

Supplementary Figure 19. Length distribution of ribosome-occupied 'sRNAs' and the other sRNAs. The longer sRNAs showed a higher frequency of ribosome occupancy.

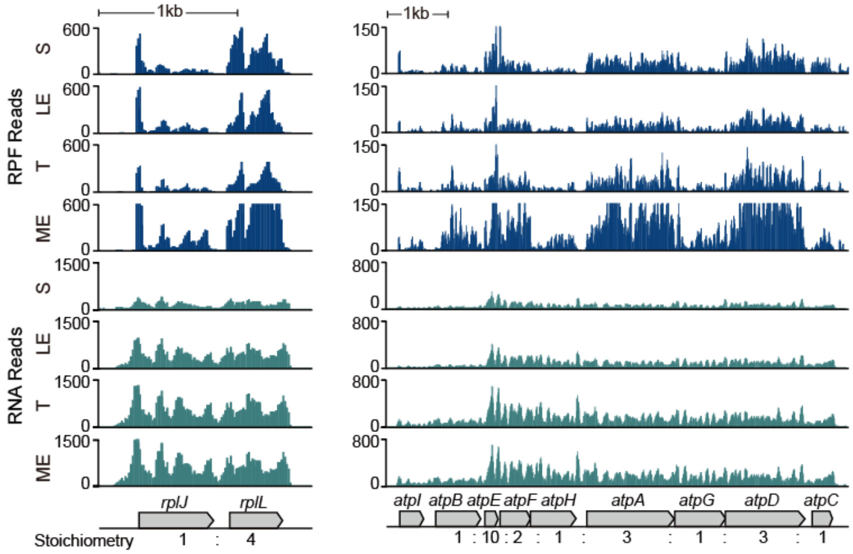

Supplementary Figure 20. Correlation between RPF levels and stoichiometry of subunits within protein complexes. RPF data for rplJL operon and ATP synthase operon show proportional relationships with their subunit stoichiometry whereas mRNA levels did not reflect this proportionality.

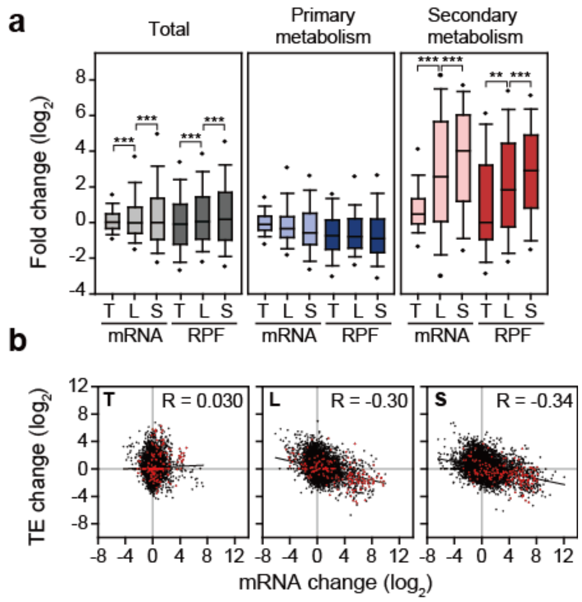

Supplementary Figure 21. Global analysis of mRNA, RPF and TE fold-change after using threshold cutoff (to remove low level ssRNA-seq and RPF expression values). The cutoff criterion for mRNA expression (=32.8) is described in legend to **Supplementary Fig. 10**. Same criterion was applied for cutoff value of RPF expression (=1.8). (**a**) Distribution of mRNA fold-change and RPF fold-change of total genes, primary metabolic genes, and secondary metabolic genes; **, $p < 0.01$; ***, $p < 0.001$ (Wilcoxon signed-rank test); **T**, fold-change between mid-exponential and transition phases; **L**, fold-change between mid- and late exponential phases; **S**, fold-change between mid-exponential and stationary phases. (**b**) Negative correlation between changes in mRNA levels and translational efficiency (TE) becomes higher at later growth phases. Red dots indicate secondary metabolic genes.

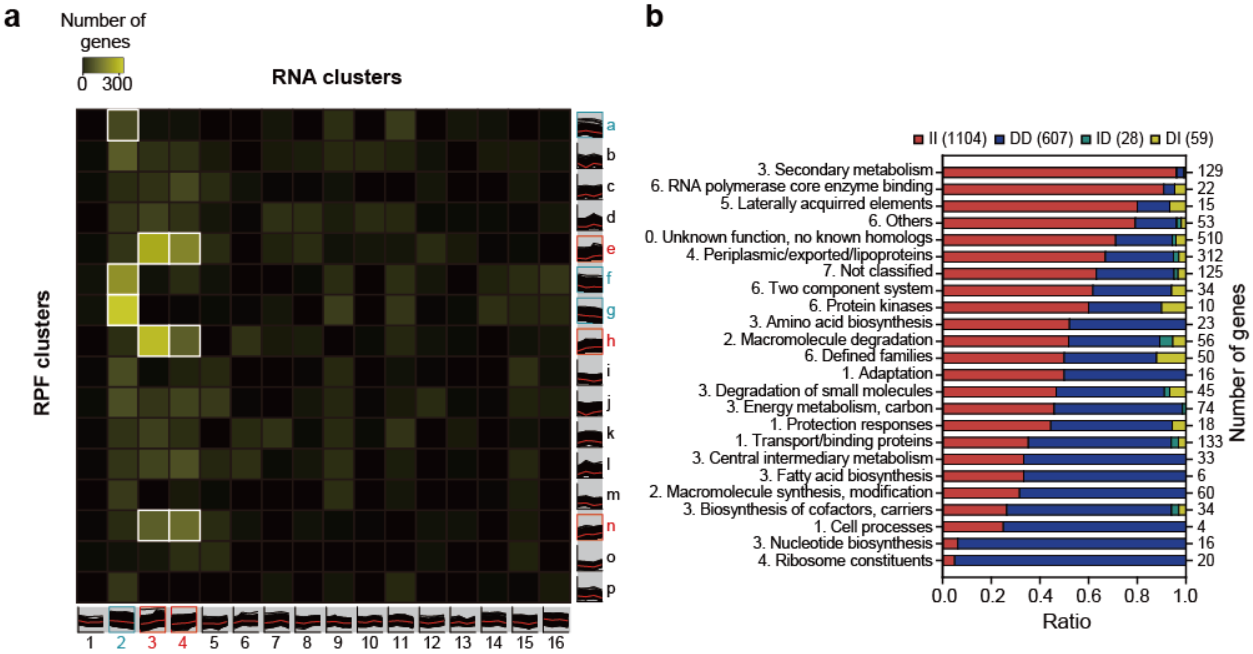

Supplementary Figure 22. Genes are clustered according to the expression patterns of mRNA (RNA; 1 – 16) or RPF (RPF; a – p). (**a**) The combination of RNA and RPF groups (16 x 16) generates 256 groups, and the number of genes included in each group is presented as a heatmap. Expression patterns of each group are plotted below (**RNA**) or to the right side (**RPF**) of the heatmap. Red boxes indicate increasing patterns, and blue boxes indicate decreasing patterns. To examine the groups having similar patterns of mRNA and RPF abundances, we designated the group for mRNA-increasing and RPF-increasing over time as **II**, and that for mRNA-decreasing and RPF-decreasing as the **DD** group. Some genes showed an anti-correlation between mRNA transcript and RPF levels: mRNA-increasing and RPF-decreasing (**ID**) group and mRNA-decreasing and RPF-increasing (**DI**) group. White boxes in the heatmap indicate **II** or **DD** groups. (**b**) Genes in each group (**II**, **DD**, **ID** and **DI**) were categorized into functional groups using the functional classification system from the Sanger Institute database. Rightmost numbers indicate the number of genes in each category. The **II** group comprised many genes in secondary metabolism along with genes related to regulation such as RNAP core enzyme binding and other transcription factors. The major categories in the **DD** group are related with cell growth, including nucleotide biosynthesis and ribosome constituents. As expected, these results showed that cells at stationary phase slow down cell division and the synthesis of most proteins. **0**, Unknown function, no known homologs; **1**, Cell processes; **2**, Macromolecule metabolism; **3**, Metabolism of small molecules; **4**, Cell envelope; **5**, Extrachromosomal; **6**, Regulation; **7**, Not classified (including putative assignments).

Supplementary Table 1. The 44 different growth conditions used for preparing a pooled RNA sample for comprehensive TSS mapping (dRNA-seq).

Condition[a]	Growth description
Liquid ME	Growth in liquid R5- medium to OD450 0.8.
Liquid T	Growth in liquid R5- medium to OD450 1.7.
Liquid LE	Growth in liquid R5- medium to OD450 2.2.
Liquid S	Growth in liquid R5- medium to OD450 2.2, followed by a further 12 hours growth.
Solid E	Growth on surface of SMMS agar medium to 27.6 mg dry weight ('mid-exponential' / rapid growth phase)
Solid T	Growth on surface of SMMS agar medium to 34.0 mg dry weight (transition phase)
Solid S	Growth on surface of SMMS agar medium to 49.0 mg dry weight (stationary phase)
Nutrient combination 1	Growth in minimal medium supplemented with 1% glucose and 0.05% ammonium chloride to OD450 0.8.
Nutrient combination 2	Growth in minimal medium supplemented with 1% glucose and 0.05% asparagine to OD450 0.8.
Nutrient combination 3	Growth in minimal medium supplemented with 1% glucose and 0.05% serine to OD450 0.8.
Nutrient combination 4	Growth in minimal medium supplemented with 1% glucose and 0.05% leucine to OD450 0.8.
Nutrient combination 5	Growth in minimal medium supplemented with 1% glucose and 0.05% glutamine to OD450 0.8.
Nutrient combination 6	Growth in minimal medium supplemented with 1% glucose and 0.05% histidine to OD450 0.8.
Nutrient combination 7	Growth in minimal medium supplemented with 1% glucose and 0.05% phenylalanine to OD450 0.8.
Nutrient combination 8	Growth in minimal medium supplemented with 1% glucose and 0.05% casamino acids to OD450 0.8.
Nutrient combination 9	Growth in minimal medium supplemented with 1% N-acetylglucosamine and 0.05% ammonium chloride to OD450 0.8.
Nutrient combination 10	Growth in minimal medium supplemented with 1% N-acetylglucosamine and 0.05% asparagine to OD450 0.8.
Nutrient combination 11	Growth in minimal medium supplemented with 1% N-acetylglucosamine and 0.05% serine to OD450 0.8.
Nutrient combination 12	Growth in minimal medium supplemented with 1% N-acetylglucosamine and 0.05% leucine to OD450 0.8.
Nutrient combination 13	Growth in minimal medium supplemented with 1% N-acetylglucosamine and 0.05% glutamine to OD450 0.8.
Nutrient combination 14	Growth in minimal medium supplemented with 1% N-acetylglucosamine and 0.05% histidine to OD450 0.8.
Nutrient combination 15	Growth in minimal medium supplemented with 1% N-acetylglucosamine and 0.05% phenylalanine to OD450 0.8.
Nutrient combination 16	Growth in minimal medium supplemented with 1% N-acetylglucosamine and 0.05% casamino acids to OD450 0.8.
Nutrient combination 17	Growth in minimal medium supplemented with 1% glycerol and 0.05% ammonium chloride to OD450 0.8.
Nutrient combination 18	Growth in minimal medium supplemented with 1% glycerol and 0.05% asparagine to OD450 0.8.

Nutrient combination 19	Growth in minimal medium supplemented with 1% glycerol and 0.05% serine to OD450 0.8.
Nutrient combination 20	Growth in minimal medium supplemented with 1% glycerol and 0.05% leucine to OD450 0.8.
Nutrient combination 21	Growth in minimal medium supplemented with 1% glycerol and 0.05% glutamine to OD450 0.8.
Nutrient combination 22	Growth in minimal medium supplemented with 1% glycerol and 0.05% histidine to OD450 0.8.
Nutrient combination 23	Growth in minimal medium supplemented with 1% glycerol and 0.05% phenylalanine to OD450 0.8.
Nutrient combination 24	Growth in minimal medium supplemented with 1% glycerol and 0.05% casamino acids to OD450 0.8.
Nutrient combination 25	Growth in minimal medium supplemented with 1% maltose and 0.05% ammonium chloride to OD450 0.8.
Nutrient combination 26	Growth in minimal medium supplemented with 1% maltose and 0.05% asparagine to OD450 0.8.
Nutrient combination 27	Growth in minimal medium supplemented with 1% maltose and 0.05% serine to OD450 0.8.
Nutrient combination 28	Growth in minimal medium supplemented with 1% maltose and 0.05% leucine to OD450 0.8.
Nutrient combination 29	Growth in minimal medium supplemented with 1% maltose and 0.05% glutamine to OD450 0.8.
Nutrient combination 30	Growth in minimal medium supplemented with 1% maltose and 0.05% histidine to OD450 0.8.
Nutrient combination 31	Growth in minimal medium supplemented with 1% maltose and 0.05% phenylalanine to OD450 0.8.
Nutrient combination 32	Growth in minimal medium supplemented with 1% maltose and 0.05% casamino acids to OD450 0.8.
NaCl shock	Growth in minimal medium to OD450 0.8 at 30°, then addition of NaCl to a final concentration of 0.5M for 1 hour.
Ethanol shock	Growth in minimal medium to OD450 0.8 at 30°, then addition of ethanol to a final concentration of 1% for 1 hour.
Heat shock (42°C)	Growth in minimal medium to OD450 0.8 at 30°, then transfer to 42° for 1 hour.
Cold shock (12°C)	Growth in minimal medium to OD450 0.8 at 30°, then transfer to 12° for 1 hour.
SDS shock	Growth in minimal medium to OD450 0.8 at 30°, then addition of SDS to a final concentration of 0.01% for 1 hour.

[a] The 44 growth conditions have been chosen to address different environments that are known to induce different gene expression pattern and different antibiotic production. Solid SMMS medium where cultures were grown to three different specific growth phases representing rapid (mid-exponential) growth, transition phase and stationary phase to capture the full developmental program of this bacterium. Note that S. coelicolor is known to undergo sporulation only when grown on solid media. The liquid media can be divided into three categories; complex media also used in ssRNA-seq and Ribo seq, different combinations of C source and N source that were used to induce different gene expression and extreme stress conditions such as ethanol, osmotic pressure, detergent (SDS), cold- and heat-shock conditions that were used to induce, for example, molecular chaperones and antibiotic production.

Supplementary Table 2. The transcription start sites associated with gene clusters encoding known secondary metabolite biosynthetic pathways.

Secondary metabolite	Start	End	Number of genes	This study	Romero et al.[a]	Vockenhuber et al.[a]
Eicosapentaenoic acid	SCO0124	SCO0129	6	0	0	0
Isorenieratene	SCO0185	SCO0191	7	1	1/2	0
Lantibiotic	SCO0267	SCO0270	4	0	0/1	0
Coelichelin	SCO0489	SCO0499	11	8	0	0
Bacteriocin	SCO0753	SCO0756	4	1	0	0
THN/flaviolin	SCO1206	SCO1208	3	0	0	0
PKS	SCO1265	SCO1273	9	1	0	0
5-Hydroxyectoine	SCO1864	SCO1867	4	4	1/2	0
Melanin	SCO2700	SCO2701	2	0	0	0
Desferrioxamine	SCO2782	SCO2785	4	1	0	0
CDA	SCO3210	SCO3249	40	11	2/3	1/1
Actinorhodin	SCO5071	SCO5092	22	10	4/4	3/4
Albaflavenone	SCO5222	SCO5223	2	0	0	0
Gray spore pigment	SCO5314	SCO5320	7	2	1/2	0
Siderophore	SCO5799	SCO5801	3	1	0	0
Prodiginine	SCO5877	SCO5898	22	8	2/3	0
Geosmin	SCO6073		1	0	0	0
SCB1	SCO6266		1	2	1/1	1/1
Coelimycin P1	SCO6273	SCO6288	16	7	1/2	2/2
Dipeptide	SCO6429	SCO6438	10	2	0	0
SapB	SCO6681	SCO6685	5	0	0	0
Hopene	SCO6759	SCO6771	13	5	2/2	0
PKS	SCO6826	SCO6827	2	1	0	0
Lantibiotic	SCO6927	SCO6932	6	0	0	0
Germicidin	SCO7221		1	2	1/1	0
PKS	SCO7669	SCO7671	3	0	0	0
Coelibactin	SCO7681	SCO7691	11	1	0	0
2-Methylisoborneol	SCO7700	SCO7701	2	0	0	0
**Total**			221	68	16/23	7/8

[a] TSSs matched with this study / TSSs identified in previous study

Supplementary Table 3. Selected gene specific primers used for 5’-Rapid amplification of cDNA ends (RACE).

Gene	Primer sequence (5' to 3')	Product length (bp)
SCO5877	GAAGTTGTACAGGCTGGGTC	221
SCO5878	GACGATGGCGATGGGTTC	243
SCO5881	TCCACCTGCACTTTCATGTC	242
SCO5887	CGAGTCCATCTCCAGCTCTT	239
SCO5888	GCCAGGTCGGAGGTGAG	224
SCO5897-antisense(1)	GGTGATCCCGGCGATGG	263
SCO5897-antisense(2)	CGGATCACCAACCACCG	229

Supplementary Table 4. Primers used for quantitative real-time PCR (qRT-PCR).

Gene	Primer sequence (5' to 3')		Product length (bp)
SCO1845	Forward	CCAACACGATCTCCAAGGGT	101
	Reverse	CCAGGTCAGGAGGTTCCA	
SCO2920	Forward	CCCAGCCCGGACAAGA	100
	Reverse	CGGATATGACCTGCTTGAGG	
SCO3005	Forward	GTCCTCTCGAAGCTCATGC	101
	Reverse	AGGTCAGCGAAGTCCTCTT	
SCO3217	Forward	TCGTTTCTTTCGACTCCCTG	105
	Reverse	GGAAGATTTTGCGCAGTGTG	
SCO3230	Forward	CTGAGAACTCTTCGGTTCGG	103
	Reverse	CAGGATCCCGTCCGGTAG	
SCO3563	Forward	CTCAAGGAAGAACGCAGGTT	103
	Reverse	AGAAGCCGAGCCTGTCA	
SCO4142	Forward	CCAACAGCAACATCAAGTGC	101
	Reverse	GACGTACTGCTTGACCCAG	
SCO4945	Forward	CACGACGTCCTGATCGACAT	100
	Reverse	CGGGGACCATCGGGAAG	
SCO5777	Forward	CAAGGAAGACAAGGCTGCG	105
	Reverse	CGCTGAATCGTCAGGTCAAT	
SCO5898	Forward	CTCTACCTGCTGCGCTTC	100
	Reverse	GGATCACCAGGACCCAGAT	

Supplementary References

1 d'Hérouel, A. F. *et al*. A simple and efficient method to search for selected primary transcripts: non-coding and antisense RNAs in the human pathogen *Enterococcus faecalis*. *Nucleic Acids Res*. **39(7)**, e46 (2011).

2 Vockenhuber, M. P. *et al.* Deep sequencing-based identification of small non-coding RNAs in *Streptomyces coelicolor*. *RNA Biol.* **8**, 468-477 (2011).

3 Romero, A. D. *et al.* A comparison of key aspects of gene regulation in *Streptomyces coelicolor* and *Escherichia coli* using nucleotide-resolution transcription maps produced in parallel by global and differential RNA-sequencing. *Mol. Microbiol.* **94**, 963-987 (2014).

4 Poralla, K., Muth, G. & Hartner, T. Hopanoids are formed during transition from substrate to aerial hyphae in *Streptomyces coelicolor* A3(2). *Fems Microbiol. Lett.* **189**, 93-95 (2000).

5 Moody, M. J., Young, R. A., Jones, S. E. & Elliot, M. A. Comparative analysis of non-coding RNAs in the antibiotic-producing *Streptomyces* bacteria. *BMC Genomics* **14**, 558 (2013).
